# Adjuvant Trastuzumab in HER2-Positive Early Breast Cancer by Age and Hormone Receptor Status: A Cost-Utility Analysis

**DOI:** 10.1371/journal.pmed.1002067

**Published:** 2016-08-09

**Authors:** William Leung, Giorgi Kvizhinadze, Nisha Nair, Tony Blakely

**Affiliations:** 1 Department of Public Health, University of Otago, Wellington, New Zealand; 2 Department of Medicine, University of Auckland, Auckland, New Zealand; Medical School, King's College London, UNITED KINGDOM

## Abstract

**Background:**

The anti–human epidermal growth factor receptor 2 (HER2) monoclonal antibody trastuzumab improves outcomes in patients with node-positive HER2+ early breast cancer. Given trastuzumab’s high cost, we aimed to estimate its cost-effectiveness by heterogeneity in age and estrogen receptor (ER) and progesterone receptor (PR) status, which has previously been unexplored, to assist prioritisation.

**Methods and Findings:**

A cost-utility analysis was performed using a Markov macro-simulation model, with a lifetime horizon, comparing a 12-mo regimen of trastuzumab with chemotherapy alone using the latest (2014) effectiveness measures from landmark randomised trials. A New Zealand (NZ) health system perspective was adopted, employing high-quality national administrative data.

Incremental quality-adjusted life-years for trastuzumab versus chemotherapy alone are two times higher (2.33 times for the age group 50–54 y; 95% CI 2.29–2.37) for the worst prognosis (ER−/PR−) subtype compared to the best prognosis (ER+/PR+) subtype, causing incremental cost-effectiveness ratios (ICERs) for the former to be less than half those of the latter for the age groups from 25–29 to 90–94 y (0.44 times for the age group 50–54 y; 95% CI 0.43–0.45). If we were to strictly apply an arbitrary cost-effectiveness threshold equal to the NZ gross domestic product per capita (2011 purchasing power parity [PPP]–adjusted: US$30,300; €23,700; £21,200), our study suggests that trastuzumab (2011 PPP-adjusted US$45,400/€35,900/£21,900 for 1 y at formulary prices) may not be cost-effective for ER+ (which are 61% of all) node-positive HER2+ early breast cancer patients but cost-effective for ER−/PR− subtypes (37% of all cases) to age 69 y. Market entry of trastuzumab biosimilars will likely reduce the ICER to below this threshold for premenopausal ER+/PR− cancer but not for ER+/PR+ cancer. Sensitivity analysis using the best-case effectiveness measure for ER+ cancer had the same result. A key limitation was a lack of treatment-effect data by hormone receptor subtype. Heterogeneity was restricted to age and hormone receptor status; tumour size/grade heterogeneity could be explored in future work.

**Conclusions:**

This study highlights how cost-effectiveness can vary greatly by heterogeneity in age and hormone receptor subtype. Resource allocation and licensing of subsidised therapies such as trastuzumab should consider demographic and clinical heterogeneity; there is currently a profound disconnect between how funding decisions are made (largely agnostic to heterogeneity) and the principles of personalised medicine.

## Abstract 2

In a Markov simulation using New Zealand health data, William Leung and colleagues assess the age- and subtype-specific cost-effectiveness of trastuzumab with breast cancer chemotherapy.

## Author Summary

**Why Was This Study Done?:**

- Early-stage human epidermal growth factor receptor 2–positive (HER2+) breast cancer tends to be more aggressive and resistant to standard chemotherapy, resulting in a poorer prognosis.
- In patients where the cancer has spread regionally, a 12-mo course of trastuzumab (Herceptin, ~NZ$67,800 or ~€31,600 at 2011 ex-factory prices) is usually added to standard chemotherapy treatment, which further reduces the risk of death by a third.
- Economic evaluations in high-income countries have found that this use of trastuzumab generally provides value for money; however, its cost-effectiveness for specific subtypes of HER2+ breast cancer (defined by estrogen receptor [ER] and progesterone receptor [PR] status) is unknown.

**What Did the Researchers Do and Find?:**

- A computer model was developed that simulated the life courses of patients from chemotherapy through to death from breast cancer or another cause, to estimate lifetime outcomes with and without trastuzumab therapy.
- In the model’s results, patients with the breast cancer subtype with the worst prognosis (ER−/PR−) receive twice the health benefit (in quality-adjusted life-years, a measure of disease burden that considers the quality and quantity of life lived) of those with the subtype with the best prognosis (ER+/PR+) for trastuzumab therapy versus chemotherapy alone.
- For the worst prognosis subtype, the health system costs required to gain a quality-adjusted life-year were half of that for the best prognosis subtype.

**What Do These Findings Mean?:**

- This study demonstrates the value of investigating cost-effectiveness by different subtypes within a disease, thus potentially allowing for a more targeted allocation of scarce health resources.
- These modelling results are not intended to act as guidelines to determine who should or should not receive treatment.

## Introduction

Between 12% and 25% of breast cancer cases are human epidermal growth factor receptor 2 (HER2) positive [1–3]. Tumours that are HER2+ tend to be more aggressive and more resistant to standard chemotherapy, resulting in a poorer prognosis [4]. Trastuzumab, a monoclonal antibody that binds the HER2 protein, was the first agent to target HER2+ breast cancer.

As an adjuvant treatment for early stage HER2+ breast cancer patients with node-positive disease or a tumour ≥ 1 cm, a 12-mo regimen of trastuzumab therapy compared with chemotherapy alone reduces the risk of death by a third (hazard ratio [HR] for overall survival [OS] = 0.66; 95% CI 0.57–0.77), although (usually reversible) cardiac dysfunction may occur [5]. This relative reduction in hazard appears to last for at least 10 y post-diagnosis [6].

At present, if patients meet left ventricular ejection fraction requirements at baseline and during treatment, clinical practice guidelines recommend a 12-mo regimen of trastuzumab, typically administered concurrently with a taxane, after prior anthracycline therapy [7–10]; efficacy data favour concurrent administration over sequential administration [5].

The optimal duration of trastuzumab therapy is unknown. The current 12-mo regimen is the result of the landmark Herceptin Adjuvant (HERA) [11], Breast Cancer International Research Group (BCIRG) 006 [12], North Central Cancer Treatment Group (NCCTG) N9831, and National Surgical Adjuvant Breast and Bowel Project (NSABP) B-31 trials [13]. In the HERA trial, 2 y of adjuvant trastuzumab was found to be not more effective than 1 y of treatment [14]. The PHARE trial compared 6 mo versus 12 mo of trastuzumab and failed to show non-inferiority of the shorter treatment regimen [15]. Other large trials comparing 9 wk, 3 mo, and 6 mo versus 12 mo of trastuzumab—regimens that are less cardiotoxic and would enable more patients to be treated for the same cost—are underway [16–18]. Until they report, a 12-mo regimen remains the standard of care.

While trastuzumab has been a breakthrough treatment for early stage breast cancer, it is a high-cost pharmaceutical; in New Zealand (NZ), administration of a 12-mo trastuzumab regimen costs NZ$74,000 (excluding confidential discounts negotiated by NZ’s national pharmaceutical agency, PHARMAC) on top of standard chemotherapy—at least according to the listed prices [19]. The UK National Institute for Health and Care Excellence (NICE) concluded that published economic evaluations have indicated that the 12-mo regimen can generally be considered cost-effective in the adjuvant setting as no study reported a base-case incremental cost-effectiveness ratio (ICER) above £30,000 (US$50,000) per quality-adjusted life-year (QALY) [20]. In most base-case analyses, the modelled patient cohort was assumed to be 50 y old (the median age) and from one of the landmark trials. However, when ICERs have been reported for older patient cohorts, age ≥ 75 y at treatment initiation, ICERs have typically been over €100,000, or US$100,000, per QALY [21–23]. Similarly, a Belgian study estimated that the incremental cost per life-year gained for a HERA cohort aged 80+ y at treatment initiation was over €450,000 for stage II breast cancers but only €57,802 for stage III tumours [24].

While those studies have highlighted the importance of heterogeneity by age (at treatment initiation) and tumour stage, no study has also investigated the cost-effectiveness by estrogen receptor (ER) and progesterone receptor (PR) status in early stage HER2+ breast cancer. A large Californian study of all the possible ER, PR, and HER2 combinations showed that patients with ER-negative subtypes have worse 5-y survival than those with HER2+ status [3].

As health system resources have to be prioritised, it is surprising that the cost-effectiveness of trastuzumab has not been more thoroughly examined by age and hormone receptor subtype. Therefore, this paper aims to estimate the cost-effectiveness of a 12-mo trastuzumab regimen, compared to standard chemotherapy alone, by the four ER/PR subtypes (ER+/PR+ has the best prognosis; ER−/PR− has the worst prognosis) and then by age of treatment initiation (age groups from 25–29 to 90–94 y) in early stage HER2+ breast cancer that has spread regionally (node positive). We hypothesised that trastuzumab therapy for women with HER2+ breast cancer may not be cost-effective for older patients, nor for the better prognosis subtypes (ER+/PR+, ER+/PR−, and ER−/PR+), and doubly so for older women with better prognosis subtypes.

## Methods

### Model

We constructed a Markov macro-simulation model (TreeAge Pro 2014, TreeAge Software) comparing the lifetime costs and effectiveness of adjuvant trastuzumab compared to chemotherapy alone for patients with node-positive HER2+ early breast cancer.

A hypothetical cohort clinically similar to those enrolled in the NSABP B-31 and NCCTG N9831 trials was considered, comprising 37% ER+/PR+, 15% ER+/PR−, 3% ER−/PR+, and 45% ER−/PR−. The trials’ concurrent trastuzumab treatment regimen most closely mirrored clinical practice in NZ, 94% of randomised patients had node-positive cancer, and the joint analysis of the two trials had the longest reported follow-up (median 8.4 y) of any of the landmark trials [6].

**Fig 1:**
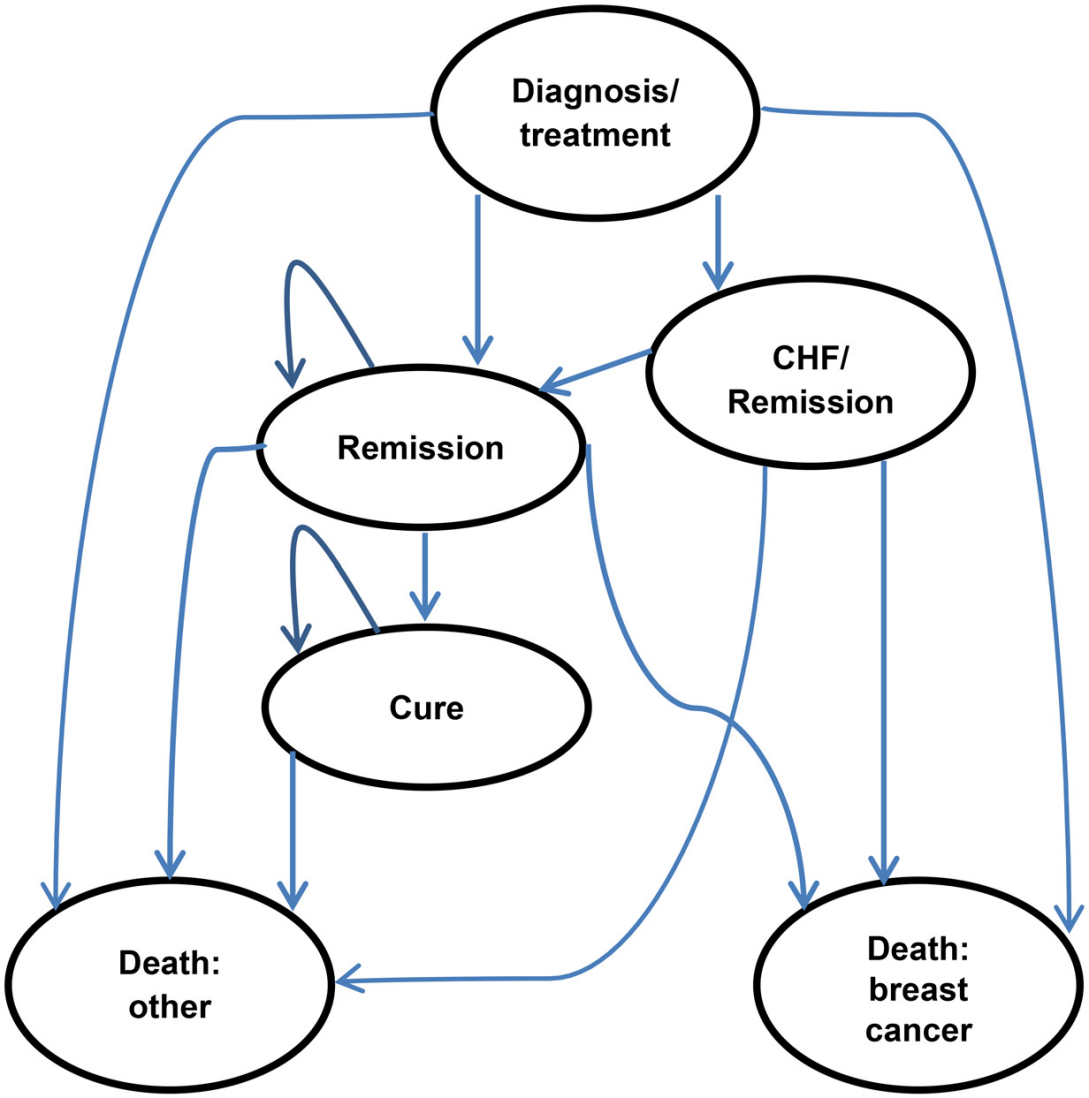
Health states in the Markov model. CHF, congestive heart failure.

The Markov model tracked patients’ transitions among mutually exclusive health states that last a fixed length of time. The six health states in the Markov model are shown in Fig 1. During each 12-mo cycle, patients accumulate QALYs and costs. Transitions occur according to input probabilities.

Patients entered the model in the post-diagnosis state, after completing the first part of adjuvant chemotherapy (treatment with an anthracycline). In the chemotherapy-alone arm, patients received only a taxane during the 4-mo initial treatment period. In the trastuzumab arm, patients received the same 4-mo taxane chemotherapy regimen plus concurrent trastuzumab for a period of 12 mo to give the required total dosage of 110 mg per kilogram of body weight.

Patients receiving adjuvant trastuzumab may incur moderate or severe congestive heart failure (CHF), as defined by New York Heart Association (NYHA) class III and IV, respectively. This adverse event causes a permanent discontinuation of trastuzumab treatment and causes changes in both costs and quality of life. The model did not include breast cancer recurrence as a state explicitly, but its impact is estimated mathematically (see S1 Text). The benefit in terms of survival from trastuzumab is assumed to last 8 y. Death from breast cancer is preceded by an 11-mo pre-terminal and a 1-mo terminal state.

### Clinical Data

The estimated incidence rates of invasive early breast cancer were calculated using New Zealand Cancer Registry data linked to census data [25]. Breast cancer mortality rates were estimated using excess mortality rate (the “extra” mortality from breast cancer, the mortality analogue of relative survival) modelling [26] on New Zealand Cancer Registry data linked to mortality data. We had excess mortality rate (EMR) equations by SEER cancer stage (i.e., local and regional) [27] from previous work [28]. However, EMRs by local or regional stage do not capture the heterogeneity in prognosis by other clinical characteristics. Prognosis within regional breast cancer varies markedly by ER/PR status, HER2 status, tumour grade, etc. [29]. In order to incorporate this heterogeneity by prognosis into our analysis, we recalibrated the existing EMR equations to match four more detailed invasive HER2+ early breast cancer subtypes, as defined by regional spread, ER status, and PR status. Briefly, EMRs were calculated for each breast cancer subtype using the relative risk (RR) of mortality, and proportionate incidence, by subtype, calibrated to give the same total EMR as observed with the NZ cancer data followed up for mortality. The RR for OS at diagnosis was taken from the joint analysis of the B-31 and N9831 trials (HR = 0.63; 95% CI 0.54–0.73) [6]. This RR was mathematically converted to RRs for breast cancer death by age (Table 1), by allowing for competing mortality from cancer and other causes. Further details on this calibration are provided in S1 Text.

**Table 1:** Key model parameters.

Category	Parameter	Best Estimate	Variation	Source
**Mortality rates**	**Probability of death from breast cancer**			EMRs for regional breast cancer from New Zealand Cancer Registry data varying by sociodemographic stratum [28]
	**Probability of death from other causes**			Mortality rates from NZ life tables incorporating heterogeneity by sociodemographic stratum [30]
**Trastuzumab-related parameters**	**HR for breast cancer death for trastuzumab versus chemotherapy alone (by age of treatment initiation)**		Log-normal distribution	N9831 and B-31 follow-up [6] adjusted from death from all causes to death from breast cancer
	Age < 40 y	0.63	SE(lnRR) = 0.078	
	Age 40–49 y	0.62	SE(lnRR) = 0.084	
	Age 50–59 y	0.56	SE(lnRR) = 0.106	
	Age ≥ 60 y	0.50	SE(lnRR) = 0.136	
	**CHF rate (NYHA class III or IV)**		Log-normal distribution	
	Trastuzumab	0.01	SE(lnRR) = 0.211	From post-anthracycline therapy in the N9831 and B-31 trials [31,32] and NZ observational data
	Chemotherapy alone	0	n/a	
**Disease model states**	**Diagnosis and treatment duration**	4 mo	n/a	Period of taxane chemotherapy
	**Pre-terminal duration**	11 mo prior to entering terminal state	n/a	Derived from Australian burden of disease models [33,34]
	**Terminal duration**	1 mo prior to death	n/a	Same as above
	**Statistical cure time-point**	20 y	n/a	Same as above
**Disability weights**	**Diagnosis and treatment disability weight**	0.194	Beta distribution; Alpha 1 = 17.57; Alpha 2 = 72.99	Derived from 2010 GBD Study adapted for NZ [33,35]
	**Pre-terminal disability weight**	0.513	Alpha 1 = 19.59; Alpha 2 = 18.60	Same as above
	**Terminal disability weight**	0.521	Alpha 1 = 18.75; Alpha 2 = 17.24	Same as above
	**Remission disability weight**	0.174^a^	Alpha 1 = 17.14; Alpha 2 = 81.34	Same as above
	**CHF disability weight**	0.088	Alpha 1 = 39.29; Alpha 2 = 401.8	Derived from 2010 GBD Study [35] and NYHA class III and IV CHF weightings from trials [36]
	**Duration of CHF**	6 mo	n/a	B-31 trial [37]
**Costs**	**Population health system costs**		Gamma distribution with SE of ±10%	Linked administrative health data for the entire NZ population by age from Ministry of Health databases [33]
	**Intervention costs**		Gamma distribution with SE of ±10%	Various Ministry of Health, District Health Board, and PHARMAC sources, as described elsewhere [38]

^a^Discounted at 20% per annum from end of year 1.

GBD Study, Global Burden of Disease Study; n/a, not applicable; SE, standard error.

The rate of CHF in the trastuzumab arm was taken from the N9831 and B-31 trials and NZ observational data. Both cardiac mortality and the rate of CHF were assumed to be those expected in the background population in the chemotherapy-alone arm. To simplify the model, in the base case all patients incurring CHF did so at 6 mo [31] and subsequently discontinued adjuvant trastuzumab, receiving half the benefit from the drug. Symptoms from CHF were assumed to be reversible, lasting 6 mo [37].

### Costs

Costs were estimated from a NZ health system perspective and are in year 2011 values (adjusted using the NZ consumer price index) [38]. A discount rate of 3% per annum was applied to both costs and benefits. All costs exclude Goods and Services Tax.

Unit costs of the components of each intervention and the number of resource units consumed are shown in Table 2. Baseline health system costs derived from the real costs for patients with regional breast cancer in NZ are included in the model. Because these costs already include the real costs for chemotherapy averaged across all patients with early breast cancer, only the incremental cost of the trastuzumab administration (and related toxicities) compared with chemotherapy alone are included in the intervention costs. Diagnosis and treatment costs include initial diagnosis and staging of breast cancer, surgery, radiotherapy, and administration of standard chemotherapy.

**Table 2:** Unit costs (2011 New Zealand dollars) for intervention.

Cost Item	2011 Unit Cost	Number of Units	Cost		Source and Notes
			Per Cycle	Total	
**Outpatient admission for trastuzumab administration**	493.83	1	493.83	5,925.96	MoH/DHBNZ Outpatient Purchase Unit national price for outpatient attendance for intravenous chemotherapy for cancer (MS02009) [39]. Includes all pharmaceuticals administered other than cancer drugs. Includes day-case treatment (including physician and nursing care, overheads, hotel costs, etc.). Based on trastuzumab being administered every 3 wk for an additional 8 mo (36 wk) after completion of taxane chemotherapy, i.e., an additional 12 cycles.
**Patient travel and accommodation**					Average distance based on incidence multiplied by each patient’s distance to hospital using census area units, described elsewhere [38].
Travel (per kilometre)	0.245	45.4	11.11	133.32	National Travel Assistance Policy reimbursement rate per kilometre for private vehicle use [40] multiplied by the average distance travelled by patients with regional cancer to nearest chemotherapy centre for the additional 12 cycles of trastuzumab [38].
Accommodation	174.00	0.038	6.78	81.36	National Travel Assistance Policy reimbursement rate for accommodation for a two-night stay [40] applied to proportion of patients with regional breast cancer travelling >100 km for the additional 12 cycles of trastuzumab [38].
Total travel and accommodation				214.68	Based on trastuzumab being administered for an additional 12 cycles.
**Chemotherapy**					
Trastuzumab	3,875.00	17.5	3,875.00	67,812.50	Unit cost for one 440-mg vial from NZ Pharmaceutical Schedule (Section H) in August 2011 [19]. Assumes no wastage and a 70-kg patient body weight for a dosage of 110 mg/kg over 52 wk.
**CHF**					
Outpatient admission				1,386.00	The outpatient cost of managing a patient with CHF for 6 mo [41].
Inpatient admission	4,567.49	0.43		1,966.78	MoH WIESNZ11 case-mix cost weights [42,43]. Inpatient admission for heart failure without catastrophic complications (F62B): cost weight = 0.9395. The number of units is further weighted by the proportion (46%) of NYHA class III and IV CHF cases hospitalised in the N9831 and B-31 trials [36].
Total CHF treatment costs				3,352.78	

Unit costs are purchasing power parity (PPP)–adjusted 2011 NZ dollars: NZ$1 = US$0.67 = €0.53 = £0.47 = ¥72.

DHBNZ, District Health Boards New Zealand; MoH, New Zealand Ministry of Health; WIESNZ11, New Zealand Weighted Inlier Equivalent Separation 2011.

To calculate the dosage of trastuzumab, we adopted the 70-kg average female body weight used by NZ pharmacists. Intervention costs were based on concurrent administration of trastuzumab every 3 wk (IV 8 mg/kg loading dose then 6 mg/kg every 3 wk) for a period of 12 mo. In addition, we considered the cardiac monitoring costs of patients receiving trastuzumab: echocardiography or multigated acquisition (MUGA) scan performed quarterly until end of treatment, as is current practice in NZ. The cost of 6 mo of clinical management of CHF was derived from a published NZ economic evaluation [41]. No additional long-term healthcare costs were assumed for patients incurring CHF.

Population health system costs are those downstream costs incurred or averted as a result of the intervention; both related and unrelated health system costs are included. The excess costs for regional breast cancer patients at different stages of their care (diagnosis/treatment, remission, and pre-terminal and terminal for those dying from cancer) were estimated using gamma regression. Following van Baal et al. [44], we separately determined the expected costs in the last 6 mo of life for those dying from causes other than cancer.

### Quality of Life

In this model, we use modified QALYs, which incorporate disability weights (DWs), rather than utilities, and allow for expected background morbidity. DWs were sourced from the 2010 Global Burden of Disease Study [35] with modification to the NZ distribution of cancers [33]; the DWs for each nonfatal model state are shown in Table 1. Expected population morbidity was allowed for by using the average ethnicity- and age-specific prevalent years of life lived with disability (pYLD) from the NZ Burden of Disease Study [33], thus limiting the maximum QALYs^DW^ that can be gained with increasing age. It is critical to note that the own population’s life table is used for QALY^DW^ calculations, rather than an “ideal” life table, as is used for calculation of disability-adjusted life-years (DALYs) in burden of disease studies, and no age weighting is used. That is, the QALYs^DW^ we estimate are standard QALYs, but using DWs to value health states.

Patients who do not die from breast cancer or other causes remain in the remission stage for a maximum of 20 y, during which time they accumulate the QALYs^DW^ associated with the remission DW. After that, they return to a state of normal health where their morbidity is the same as that of others of the same age and ethnicity who have not experienced breast cancer. Those dying of breast cancer assume the higher DWs of the pre-terminal phase for the final 11 mo prior to the terminal phase and of the terminal phase for the last month of life. No additional DW is assigned prior to death for those dying of other causes, but costs are adjusted for the higher costs of the last 6 mo of life.

### Analyses

ICERs and incremental QALYs^DW^ and costs were calculated for each of the four individual ER/PR subtypes, and the values for the subtypes were pooled together, by 5-y age group from 25–29 to 90–94 y, for adjuvant trastuzumab compared to chemotherapy alone. We averaged across the heterogeneity in ethnicity and deprivation (socioeconomic stratum) in all results.

Monte Carlo simulation was used to address parameter uncertainty, with 2,000 draws from input parameters based on the following distributions: log-normal distribution for the HR for OS; beta distributions for the proportions experiencing DWs; gamma distributions for costs (see Table 1). Cost-effectiveness acceptability curves were calculated for each ER/PR subtype.

In sensitivity analyses, we reran models for a range of scenarios to assess the impact of structural assumptions: discount rate set at 0% or 6% per annum; cost of trastuzumab decreased by 30% (from 2011 NZ$67,812 to NZ$47,469) to mimic biosimilar competition; the benefit in terms of survival from trastuzumab (i.e., the effect size) was extended to 20 y; HR for OS reduced (from 0.63 to 0.49) for the three better prognosis subtypes (ER+/PR+, ER+/PR−, and ER−/PR+) as a proxy for treatment-effect heterogeneity; benefit received on 6-mo discontinuation of trastuzumab increased to 75%; all DWs and pYLD rates removed (to therefore generate life-years gained rather than QALYs^DW^); and unrelated health system offset costs removed.

We also conducted a range of one-way sensitivity analyses, using the 2.5th and 97.5th percentile values of input parameters (expected values only; no uncertainty about input parameters), to assess which input parameters contributed the most to the uncertainty in the model incremental QALY^DW^ and cost outputs and ICERs. This economic evaluation was conducted in accordance with the Consolidated Health Economic Evaluation Reporting Standards (CHEERS) guidelines [45].

## Results

### Base Case

**Fig 2:**
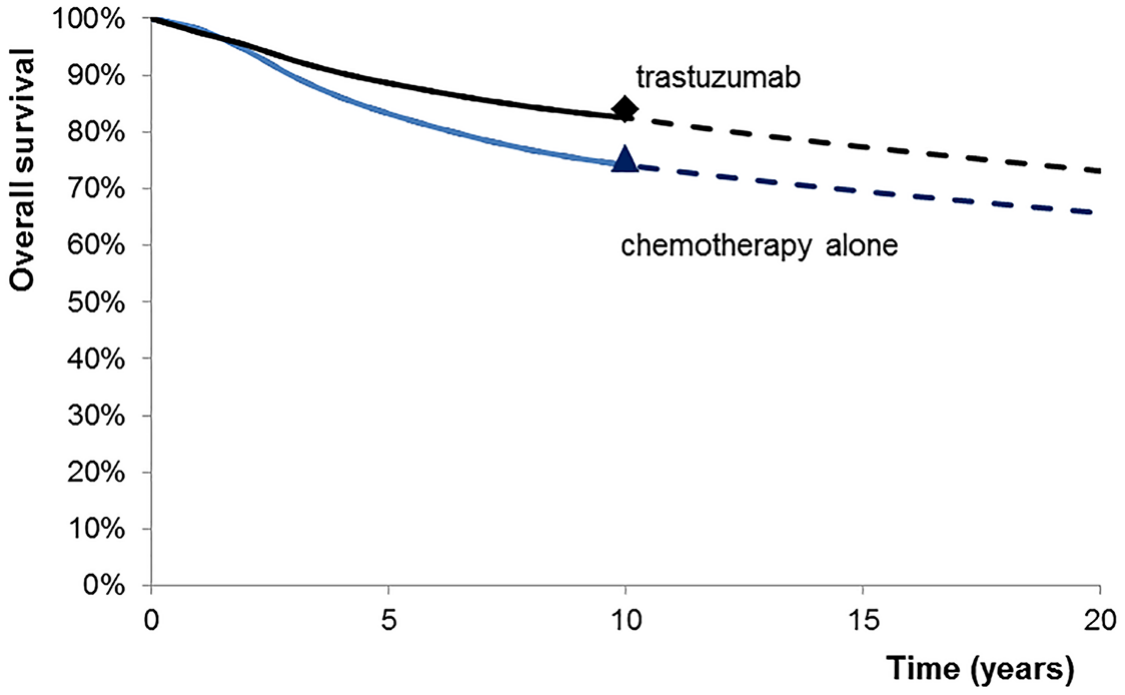
Overall survival. Curves indicate model outputs. Diamond and triangle markers indicate reported 10-y OS from the N9831 and B-31 trials [6].

The model calculated that adjuvant trastuzumab improved 20-y OS from 65% to 73% and 10-y OS from 74% to 82.5% in the median age group 50–54 y with the same hormone receptor subtype distribution as in the N9831 and B-31 clinical trials [13]. Model-derived survival curves did not differ from the results of the N9831 and B-31 clinical trials at 10 y (Fig 2) [6]. For the age group 50–54 y, adjuvant trastuzumab resulted in 0.74 (for the ER+/PR+ [best prognosis] subtype) to 1.72 (for the ER−/PR− [worst prognosis] subtype) QALYs^DW^ gained per patient treated (Table 3); patients with ER−/PR− cancer benefited from 2.33 (95% CI 2.29–2.37) times greater QALY gains than ER+/PR+ patients. Incremental costs per patient ranged from NZ$72,581 (ER+/PR+) to NZ$73,771 (ER−/PR−). There was little variation in incremental costs by age group or subtype, with cost-effectiveness driven mainly by differences in QALY^DW^ gains. This resulted in ICERs from below NZ$45,000 (ER−/PR−) to nearly NZ$100,000 (ER+/PR+) per QALY^DW^.

**Table 3:** Base-case incremental QALYs^DW^ and costs (2011 New Zealand dollars) per woman treated and ICERs (2011 New Zealand dollars), by age group and subtype.

Output by Age Group	ER+/PR+	ER+/PR−	ER−/PR+	ER−/PR−	Pooled
**Incremental QALY**					
25–44 y	0.98	1.28	1.93	2.09	1.55
45–54 y	0.73	0.98	1.55	1.70	1.23
55–64 y	0.76	1.00	1.55	1.69	1.24
65–74 y	0.72	0.94	1.39	1.49	1.12
75–84 y	0.42	0.54	0.81	0.87	0.65
≥85 y	0.19	0.25	0.39	0.42	0.31
**Incremental cost**					
25–44 y	71,770	71,783	71,492	71,304	71,554
45–54 y	72,370	72,656	73,144	73,202	72,811
55–64 y	73,059	73,609	74,624	74,791	73,968
65–74 y	72,911	73,504	74,401	74,469	73,746
75–84 y	69,279	69,250	68,648	68,307	68,818
≥85	62,857	62,482	61,219	60,753	61,805
**ICER**					
25–44 y	73,529	56,181	37,003	34,206	46,151
45–54 y	98,687	74,469	47,272	43,160	59,319
55–64 y	96,680	73,735	48,117	44,293	59,674
65–74 y	102,266	79,335	54,172	50,564	65,751
75–84 y	170,176	130,817	87,237	80,857	105,894
≥85 y	337,945	255,681	162,962	148,919	198,365

**Fig 3:**
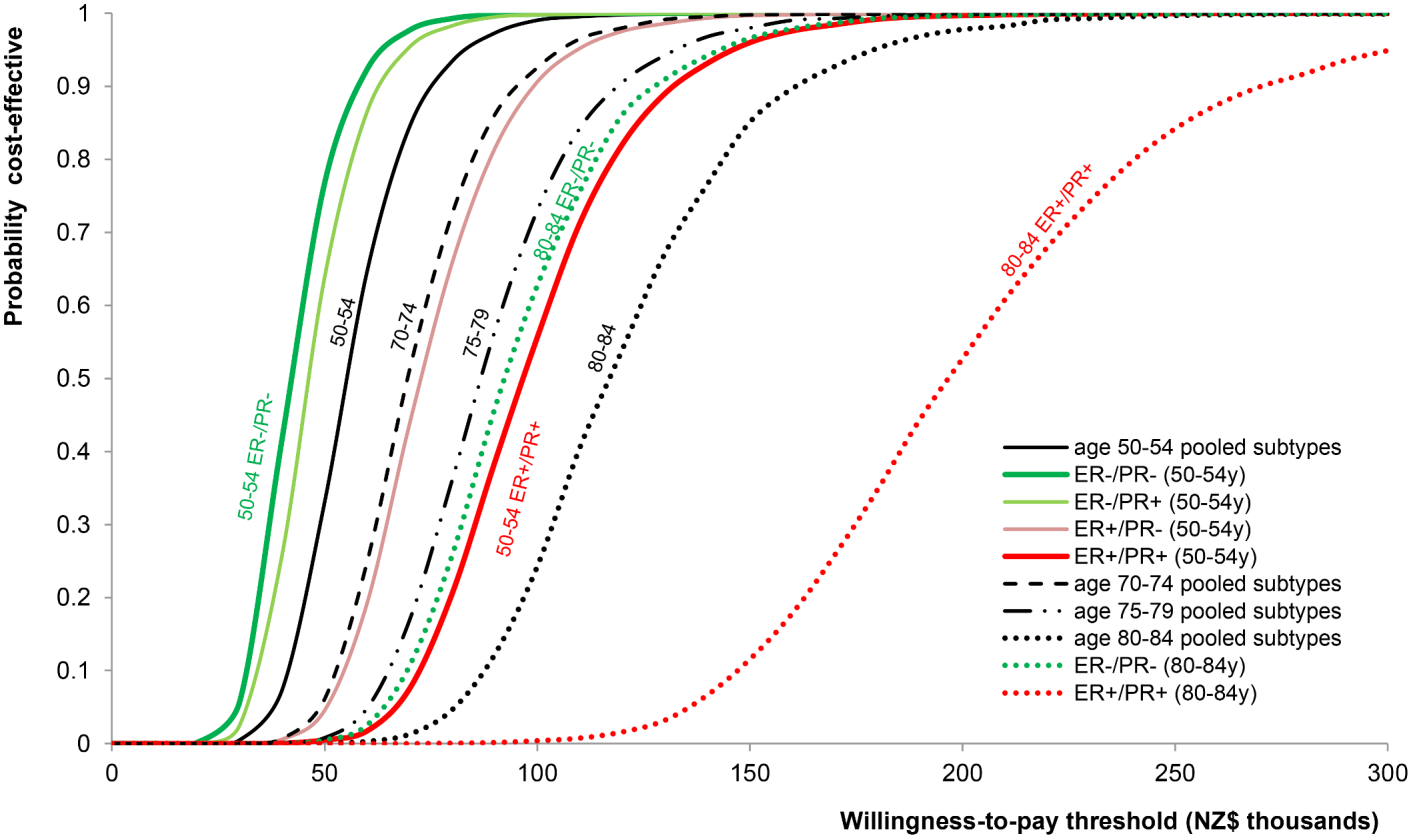
Cost-effectiveness acceptability curves.

Pooling the subtypes for the age group 50–54 y, using subtype weighting from the N9831 and B-31 trials and NZ ethnicity and deprivation weighting, gives an incremental 1.31 QALYs^DW^ gained and an incremental cost of NZ$73,379, with an ICER of NZ$56,050. At willingness-to-pay thresholds of NZ$45,000 and NZ$90,000, there is a 0.182 and 0.973 probability, respectively, that trastuzumab would be cost-effective (Fig 3). As the ICER for the worst prognosis (ER−/PR−) subtype is less than half (0.44 times; 95% CI 0.43–0.45) that for the best prognosis (ER+/PR+) subtype, the former has a 24% lower and the latter a 75% higher ICER than the estimate for the pooled subtypes.

In general, trastuzumab cost below NZ$45,000 per QALY^DW^ gained for the two ER− subtypes up to age 70 y. It was less cost-effective (below NZ$90,000 per QALY^DW^ gained) for the two ER− subtypes between the ages 70 and 79 y and for the two ER+ subtypes up to age 70 y. Full results by 5-y age group are available in S3 Table.

### Sensitivity Analyses

**Fig 4:**
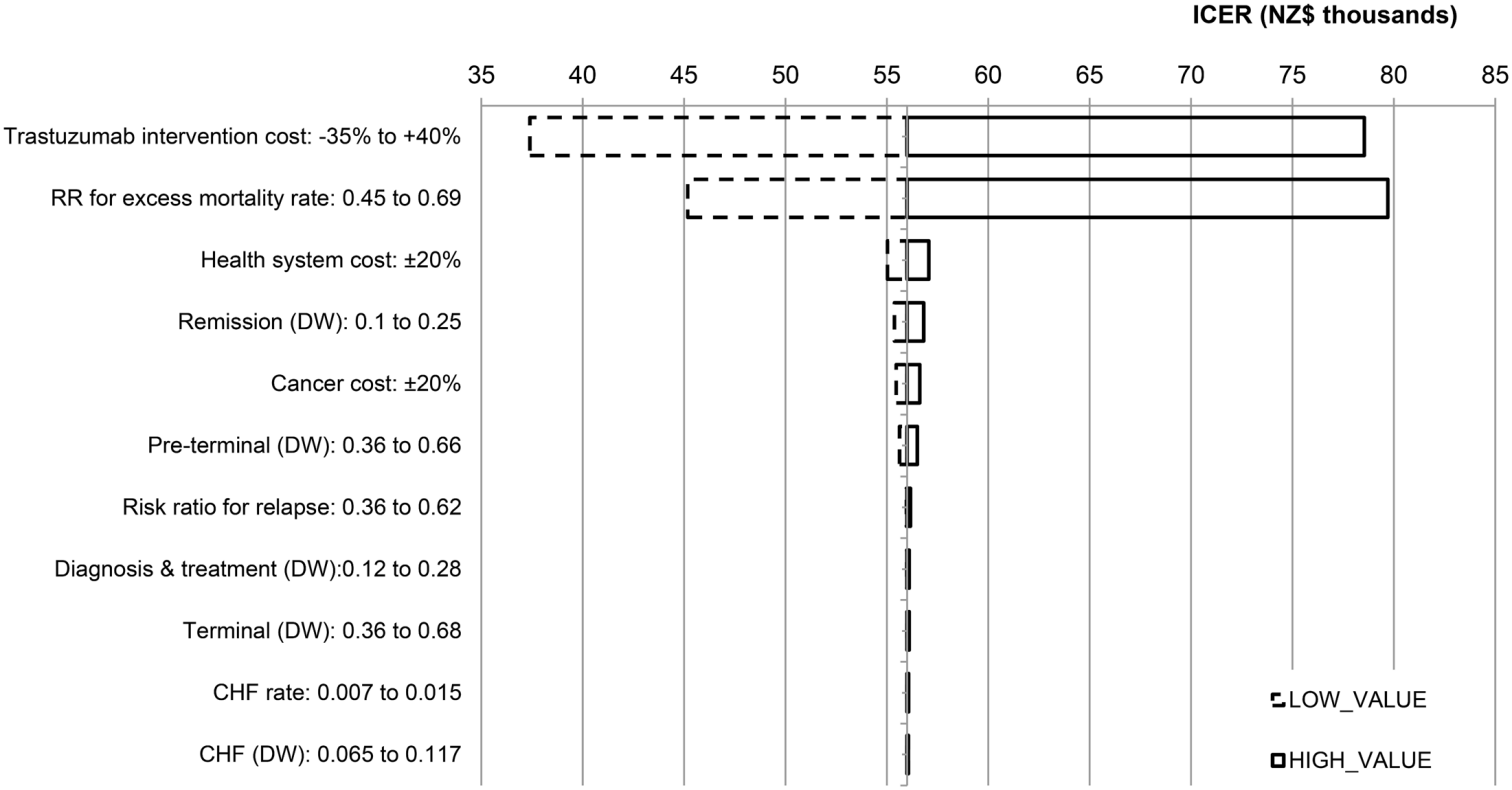
One-way sensitivity analysis: tornado plot for pooled subtypes for the age group 50–54 y.

**Fig 5:**
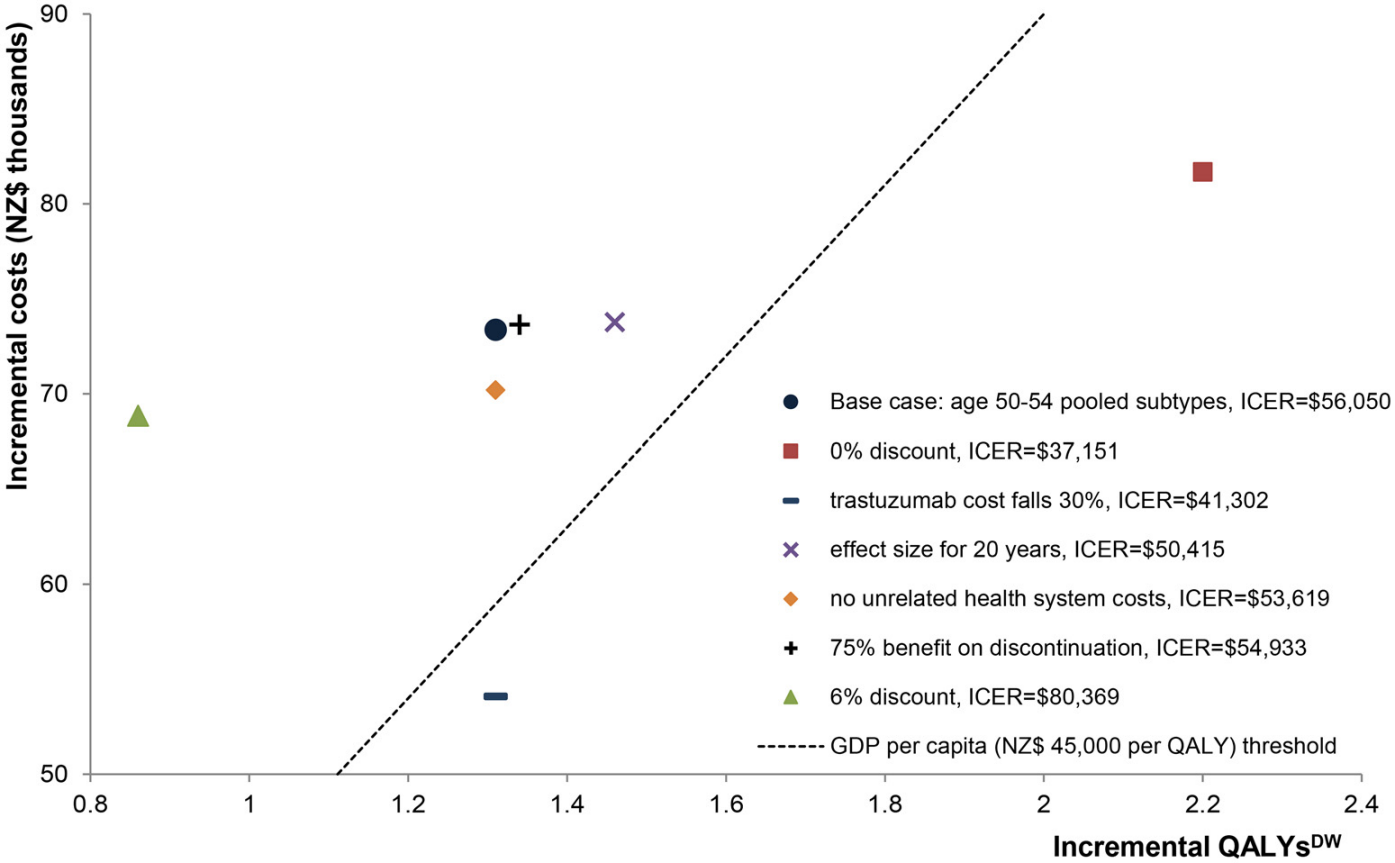
Scenario analyses: cost-effectiveness plane for the age group 50–54 y. GDP, gross domestic product.

Focusing on the median age group 50–54 y [41] weighted by subtype, ethnicity, and deprivation, the one-way sensitivity analysis (Fig 4) shows that the main drivers of uncertainty in the ICER are uncertainties in the cost of trastuzumab and the risk ratio for EMRs. Other input parameters had little impact. In scenario analyses (Fig 5), discounting had the largest impact, with a 0% discount rate giving an ICER of NZ$37,200. The cost of trastuzumab decreasing by 30% had the next largest impact, with an ICER of NZ$41,300 (full results in S7 Table; summarised in Table 4), followed by the survival benefit from trastuzumab being extended to 20 y (ICER: NZ$50,400). Simulating treatment-effect heterogeneity (HR = 0.49) for the three better prognosis subtypes increased incremental QALYs^DW^ by 41% to 47% over the base case, leading to similar cost-effectiveness results as the cost of trastuzumab decreasing by 30% (full results in S6 Table). The remaining scenarios had a minor effect. For the scenario of removing all DWs and pYLD rates, trastuzumab provided a 20.2-mo survival advantage, resulting in an incremental cost per life-year gained of NZ$43,500 (not shown in Fig 5).

**Table 4:** Scenario analysis: cost of trastuzumab decreases by 30%.

Age Group	ICER (2011 NZ Dollars) by Subtype				
	ER+/PR+	ER+/PR−	ER−/PR+	ER−/PR−	Pooled
25–44 y	53,607	41,024	27,100	25,065	33,734
45–54 y	72,143	54,581	34,850	31,864	43,587
55–64 y	71,024	54,386	35,800	33,024	44,187
65–74 y	75,379	58,753	40,504	37,886	48,892
75–84 y	124,766	96,235	64,653	60,032	78,177
≥85 y	246,127	186,488	119,282	109,108	144,974

## Discussion

One of the strengths of this study is that it is, as far as we are aware, the first to explore the cost-effectiveness of adjuvant trastuzumab in early stage HER2+ breast cancer by heterogeneity in ER/PR subtype and age. This is an important development in the literature as ER status can matter more than HER2 status for breast cancer survival [3]. Our findings suggest that incremental QALYs^DW^ (henceforth QALYs) for trastuzumab versus chemotherapy alone are between 2.1 to 2.3 times higher for the worst prognosis (ER−/PR−) subtype compared to the best prognosis (ER+/PR+) subtype, causing ICERs for the former to be less than half those of the latter.

We found that the cost-effectiveness of trastuzumab varies markedly by both age and hormone receptor status. Strictly applying a “rule of thumb” cost-effectiveness threshold of gross domestic product per capita (NZ$45,000 [2011 PPP-adjusted: US$30,300; €23,700; £21,200]), our NZ study suggests that trastuzumab may *not* be cost-effective for patients with ER+ node-positive HER2+ breast cancer (approximately 61% of expected node-positive HER2+ early breast cancer patients) [3], and that trastuzumab is highly cost-effective up to the age group 40–44 y for ER−/PR+ breast cancer (3% of cases) [3] and up to the age group 65–69 y for ER−/PR− breast cancer (37% of cases) [3]. If the cost of trastuzumab was 30% lower (NZ$47,469 for a 12-mo course), such as in the launch price of the trastuzumab biosimilar (Herzuma) approved in South Korea, then trastuzumab’s cost-effectiveness remains unchanged for ER+/PR+, is now cost-effective up to the age group 40–44 y for ER+/PR−, and is cost-effective up to the age group 70–74 y for both the ER−/PR+ and ER−/PR− subtypes (Table 4).

At this point, we feel that it is important to make it clear that these calculations are intended to illustrate how cost-effectiveness varies with different parameters, rather than to act as guidelines to determine who should or should not receive treatment; for instance, oncologists have to consider patient comorbidities, wishes, toxicities, and other factors. Our analysis adds to a growing body of examples of how incorporating heterogeneity in cost-utility analyses can result in more informed decision making. Ignoring this heterogeneity risks masking that an intervention that is cost-effective on average for a population may not be so for specific population subgroups (e.g., by age or disease severity), or vice versa, resulting in suboptimal resource allocation [46].

For example, in patients aged 60–64 y, the average cost-effectiveness of trastuzumab across all HER2+ early invasive breast cancer with regional spread was estimated at NZ$54,316 per QALY. This is not cost-effective by our arbitrary NZ$45,000 per QALY threshold, so it is possible that a policy-maker may decide not to fund trastuzumab for that age group at all. Although this example is rather contrived—as trastuzumab is typically funded for patients in high-income countries for this age group—it does demonstrate that if heterogeneity in baseline prognosis was incorporated into the economic evaluation, a policy-maker may consider funding trastuzumab for the subgroup of HER2+ breast cancer patients with the poorer prognosis (ER−) subtypes (as it is more cost-effective).

There is already good consensus on the value of incorporating heterogeneity in economic evaluations [47]. It allows different decisions to be made for dissimilar subgroups, with the potential to deliver greater population health gains and cost savings [48]. In spite of this, economic evaluations often pay limited attention to heterogeneity for reasons already well identified [46,49].

We included heterogeneity by clinical characteristics (prognosis) and patient demographics (age at treatment initiation). There was inadequate evidence to suggest significant treatment-effect heterogeneity [6]: it is biologically possible but remains contested [50]. Our four breast cancer subtypes are based on characteristics routinely identified as part of breast cancer staging and are therefore both biologically and clinically meaningful. However, Sculpher [46] commented that it may not be considered acceptable to use age to define subgroups if there is no age effect on the treatment or the disease, as is the case in this study.

Our ICERs for the subtypes combined are comparable to those from previous international research. For example, the cost-effectiveness estimate for the age group 50–54 y weighted by subtype from this model, NZ$56,050 per QALY, is similar to values from a number of previously conducted economic evaluations using the N9831 and B-31 trial data, which, when adjusted to 2011 NZ dollars, range from NZ$15,489 [21] to NZ$73,643 [51] per QALY in the base case. Base-case incremental QALYs from those studies varied from 1.42 [51] to 1.7 [52], if the study with a 15-y time horizon was excluded [21]. Our study’s 1.31 incremental QALYs falls below those estimates due to our incorporation of background morbidity.

In Italy and the US, Liberato et al. [21] found that trastuzumab was cost-effective for patients up to age 70 y but not for those older; that is, at age 80 y under two different scenarios, ICERs were €102,628 (2005 values; 2011 NZ$218,609) and US$112,587 (2005 values; 2011 NZ$207,841) per QALY. A similar ICER was reported by a Belgian study [23] based on the HERA trial. These ICERs, where all subtypes are aggregated, are 70% to 80% higher than the pooled subtype estimate for the age group 80–84 y in this study, at 2011 NZ$119,142 (ranging from NZ$93,369 [ER−/PR−] to NZ$198,669 [ER+/PR+]). While their results suggest that trastuzumab treatment may not be cost-effective for this older age group, incorporating the latest evidence on effectiveness and the introduction of trastuzumab biosimilars (the patent on Herceptin in the EU expired in 2014) is likely to make treatment more cost-effective for patients with the poorer prognosis (ER−) subtypes. The only previous analysis from a NZ perspective was a PHARMAC technology assessment report [41,53]. This report’s ICER estimate for the 12-mo concurrent treatment regimen, incorporating a confidential trastuzumab price discount, was in the range NZ$ $35,000 to NZ$50,000 (2008 values; 2011 NZ$38,171 to NZ$54,530) per QALY. A key deficiency of most previous analyses is the failure to explicitly model age and subtype; ICERs vary profoundly by age and subtype and, in our view, should be considered in resource allocation and licensing of subsidised therapies such as trastuzumab.

Reimbursement for adjuvant trastuzumab in node-positive HER2+ early breast cancer is not generally controversial in high-income countries, and there is evidence to suggest that there is a higher willingness to pay for cancer treatments; for instance, the UK government established the Cancer Drugs Fund in 2010 to pay for cancer treatments that have, among other things, been rejected by NICE under its reference case analysis. However, in low- and middle-income countries, where breast cancer incidence is increasing [54], financing access to this treatment is likely to place a significant burden on constrained health resources [55,56]. We hope that our heterogeneity analysis will assist in prioritisation there.

### Strengths and Limitations

Strengths of this model include the wealth of epidemiological data in NZ upon which the model was able to draw, including analyses by heterogeneity and population health system costs, allowing inclusion of both related and unrelated costs throughout the patients’ lifetimes.

QALYs^DW^ were used as the measure of health consequences in this analysis. Our analyses could be repeated with QALYs using the many available sets of utilities that have been obtained from breast cancer patients, but these utilities are likely to have a negligible influence on the ICER, given the trivial impact of the uncertainty in DWs shown in the tornado plot (Fig 4).

EMR ratios (EMRRs) were calibrated by age of treatment initiation using the latest updated HR for OS (HR = 0.63; 95% CI 0.54–0.73) from the N9831 and B-31 trials [6]; further details are available in S1 Text. We show in this paper that it is possible to calibrate the model to these heterogeneous strata. Whilst the calibration may not be perfect, such examination of heterogeneity undoubtedly yields a more thorough analysis than simply presenting pooled averages. Note that we assumed that within a particular age group the effect of trastuzumab is homogeneous across ER/PR strata and that differences in cost-effectiveness are driven simply by the different baseline survival rates in these groups.

A limitation of this approach is that the HR (used for the EMRRs) was derived from an intention-to-treat analysis where 20% of patients in the N9831 and B-31 trials’ chemotherapy-alone arms crossed over to the trastuzumab arms once the interim results were published, as it would not have been ethical to withhold a more effective treatment [6]. The HR from the Cochrane meta-analysis [5] (HR = 0.64; 95% CI 0.53–0.76) used data only from before any such crossover occurred, therefore precluding the possibility of a longer follow-up. Thus, the HR and EMRRs used in our model are likely to underestimate trastuzumab’s true treatment effect.

Noncardiac side effects of trastuzumab therapy were not included in the model, as sensitivity analyses in previous economic evaluations showed that they make little difference to cost-effectiveness. There is recent growing confidence in the cardiac safety of trastuzumab [37], and cardiac monitoring requirements are moving towards a less cautious approach. Temporary interruptions to trastuzumab treatment have not been modelled, as they are likely to have a small impact (on costs) as patients complete the regimen.

This analysis was modelled to occur within the current structure of the NZ health system. The regimens used are from international guidelines and are likely to be largely similar in other countries. The cost-effectiveness of various strategies used to test HER2 status, which is out of the scope of this model, has been reported previously [22,57]. A large European survey [58] reported the average (ex-factory) price of trastuzumab to be €616 per 150-mg vial in 2011, with a low of €480 in the UK and a high of €750 in Switzerland. External price referencing was the most commonly used (23 of 27 countries) price control procedure, where the price of a medicine is set on the basis of the price in one or several countries. The US Medicare payment allowance limit for 150 mg of trastuzumab in 2011 was €850 (US$1,087 adjusted for PPP) [59]. Cost in NZ was €710 (NZ$1,350 adjusted for PPP), which would place it within the above range; thus, we believe our results are generalisable to high-income countries. No information on official or commercial discounts and rebates was available in Leopold et al.’s European survey [58] or in NZ. For comparison in a middle-income country, a 150-mg vial of Roche’s off-brand trastuzumab, Herclon, costs 26,000 rupees in India [60]. This is equivalent to €1,345 after adjusting for PPP, double the cost in Europe.

DWs in our model are from the 2010 Global Burden of Disease Study, with only minor adaptation for NZ, and are thus internationally valid. The calibration of our model output OS versus the latest reported (10-y) OS from the N9831 and B-31 trials [6] shows a close match.

### Future Work

The present economic evaluation can be improved upon in the future. Heterogeneity can be further explored by the number of involved nodes, tumour size/grade, and cardiovascular risk factors. Although we used the latest available follow-up effectiveness measures from the pivotal trials and supplemented this with local NZ observational data, several shorter-regimen trials are due to report their results, as discussed previously. A meta-analysis of such trials will inform the decision regarding the optimal duration of trastuzumab therapy.

The patent on Herceptin expired in 2014 in the EU and will expire in the US in 2019. Although a trastuzumab biosimilar has not yet been approved via the EU centralised procedure, reduced-cost trastuzumab biosimilars (Hertraz from Mylan, and Herzuma from Celltrion) have been approved in other jurisdictions, and several other pharmaceutical companies have trastuzumab biosimilars in the pipeline [61]. While the expected lower cost of biosimilars may make trastuzumab more cost-effective in previously less-viable patient subgroups, cost pressures are likely to continue as new treatments are being trialled for HER2+ early breast cancer patients. For example, intravenous pertuzumab (administered with trastuzumab and standard chemotherapy) is currently being evaluated in the adjuvant context in patients with HER2+ early breast cancer in the APHINITY trial [62]. Finally, this study demonstrates that cost-utility analysis should consider demographic and clinical heterogeneity. It is ironic that in the age of personalised medicine being invoked to improve patient outcomes, we do not more explicitly consider heterogeneity in deciding what to fund.

## Supporting Information

S1 Fig Base-case cost-effectiveness acceptability curves: age 40–44 y by subtype. (TIF)

S2 Fig Base-case cost-effectiveness acceptability curves: age 60–64 y by subtype. (TIF)

S3 Fig Base-case cost-effectiveness acceptability curves: age 70–74 y by subtype. (TIF)

S4 Fig Base-case cost-effectiveness acceptability curves: age 80–84 y by subtype. (TIF)

S1 File Consolidated Health Economic Evaluation Reporting Standards (CHEERS) checklist. (PDF)

S1 Table Five-year relative survival ratio for the breast cancer subtypes in the model. (DOCX)

S2 Table Deriving intercept adjustments (and thus EMRs) for the four breast cancer subtypes. (DOCX)

S3 Table Base-case incremental QALYs and costs (NZ dollars) per woman treated and ICERs. (DOCX)

S4 Table Base-case DEP I (least deprived socioeconomic tertile) results. (DOCX)

S5 Table Base-case DEP III (most deprived socioeconomic tertile) results. (DOCX)

S6 Table Treatment-effect heterogeneity (HR = 0.49) scenario for the three better prognosis subtypes (ER+/PR+, ER+/PR−, and ER−/PR+). (DOCX)

S7 Table Scenario with trastuzumab cost decreased by 30%. (DOCX)

S1 Text EMR and HR calibration information. (DOCX)

## Abbreviations

DW: disability weight
EMR: excess mortality rate
EMRR: excess mortality rate ratio
ER: estrogen receptor
CHF: congestive heart failure
HERA: Herceptin Adjuvant
HR: hazard ratio
ICER: incremental cost-effectiveness ratio
NCCTG: North Central Cancer Treatment Group
NICE: UK National Institute for Health and Care Excellence
NSABP: National Surgical Adjuvant Breast and Bowel Project
NYHA: New York Heart Association
NZ: New Zealand
OS: overall survival
PPP: purchasing power parity
PR: progesterone receptor
pYLD: prevalent years of life lived with disability
QALY: quality-adjusted life-year
RR: relative risk

## References

[1] Lund MJ, Butler EN, Hair BY, Ward KC, Andrews JH, Oprea‐Ilies G, et al Age/race differences in HER2 testing and in incidence rates for breast cancer triple subtypes. Cancer. 2010;116:2549–2559. 10.1002/cncr.25016 20336785

[2] Royal Australasian College of Surgeons. BreastScreen Aotearoa annual report 2012: early and locally advanced breast cancer diagnosed in New Zealand patients in 2012. East Melbourne (Australia): Royal Australasian College of Surgeons; 2014.

[3] Parise CA, Bauer KR, Brown MM, Caggiano V. Breast cancer subtypes as defined by the estrogen receptor (ER), progesterone receptor (PR), and the human epidermal growth factor receptor 2 (HER2) among women with invasive breast cancer in California, 1999–2004. Breast J. 2009;15:593–602. 10.1111/j.1524-4741.2009.00822.x 19764994

[4] Burstein HJ. The distinctive nature of HER2-positive breast cancers. N Engl J Med. 2005;353:1652–1654. 16236735 10.1056/NEJMp058197

[5] Moja L, Tagliabue L, Balduzzi S, Parmelli E, Pistotti V, Guarneri V, et al Trastuzumab containing regimens for early breast cancer. Cochrane Database Syst Rev. 2012;4:CD006243 10.1002/14651858.CD006243.pub2 22513938 PMC6718210

[6] Perez EA, Romond EH, Suman VJ, Jeong J-H, Sledge G, Geyer CE, et al Trastuzumab plus adjuvant chemotherapy for human epidermal growth factor receptor 2–positive breast cancer: planned joint analysis of overall survival from NSABP B-31 and NCCTG N9831. J Clin Oncol. 2014;32:3744–3752. 10.1200/JCO.2014.55.5730 25332249 PMC4226805

[7] Senkus E, Kyriakides S, Penault-Llorca F, Poortmans P, Thompson A, Zackrisson S, et al Primary breast cancer: ESMO clinical practice guidelines for diagnosis, treatment and follow-up. Ann Oncol. 2013;24(Suppl 6):vi7–vi23. 10.1093/annonc/mdt284 23970019

[8] National Comprehensive Cancer Network. NCCN guidelines for patients: stage I and II breast cancer. Fort Washington (Pennsylvania): National Comprehensive Cancer Network; 2014.

[9] National Comprehensive Cancer Network. NCCN guidelines for patients: stage III breast cancer. Fort Washington (Pennsylvania): National Comprehensive Cancer Network; 2014.

[10] National Breast Cancer Tumour Standards Working Group. Standards of service provision for breast cancer patients in New Zealand—provisional. Wellington: Ministry of Health; 2013.

[11] Piccart-Gebhart MJ, Procter M, Leyland-Jones B, Goldhirsch A, Untch M, Smith I, et al Trastuzumab after adjuvant chemotherapy in HER2-positive breast cancer. N Engl J Med. 2005;353:1659–1672. 16236737 10.1056/NEJMoa052306

[12] Slamon D, Eiermann W, Robert N, Pienkowski T, Martin M, Press M, et al Adjuvant trastuzumab in HER2-positive breast cancer. N Engl J Med. 2011;365:1273–1283. 10.1056/NEJMoa0910383 21991949 PMC3268553

[13] Romond EH, Perez EA, Bryant J, Suman VJ, Geyer CE Jr, Davidson NE, et al Trastuzumab plus adjuvant chemotherapy for operable HER2-positive breast cancer. N Engl J Med. 2005;353:1673–1684. 16236738 10.1056/NEJMoa052122

[14] Goldhirsch A, Gelber RD, Piccart-Gebhart MJ, de Azambuja E, Procter M, Suter TM, et al 2 years versus 1 year of adjuvant trastuzumab for HER2-positive breast cancer (HERA): an open-label, randomised controlled trial. Lancet. 2013;382:1021–1028. 10.1016/S0140-6736(13)61094-6 23871490

[15] Pivot X, Romieu G, Debled M, Pierga J-Y, Kerbrat P, Bachelot T, et al 6 months versus 12 months of adjuvant trastuzumab for patients with HER2-positive early breast cancer (PHARE): a randomised phase 3 trial. Lancet Oncol. 2013;14:741–748. 10.1016/S1470-2045(13)70225-0 23764181

[16] Finnish Breast Cancer Group. The Synergism Or Long Duration (SOLD) Study. ClinicalTrials.gov NCT00593697. 2008 Jan 3 [cited 21 Jul 2015]. US National Institutes of Health. Available: https://www.clinicaltrials.gov/ct2/show/NCT00593697.

[17] Guarneri V, Frassoldati A, Bruzzi P, D’Amico R, Belfiglio M, Molino A, et al Multicentric, randomized phase III trial of two different adjuvant chemotherapy regimens plus three versus twelve months of trastuzumab in patients with HER2-positive breast cancer (Short-HER Trial; NCT00629278). Clin Breast Cancer. 2008;8:453–456. 10.3816/CBC.2008.n.056 18952561

[18] Earl HM, Cameron DA, Miles D, Wardley AM, Ogburn E, Vallier A, et al, editors. PERSEPHONE: duration of trastuzumab with chemotherapy in women with HER2-positive early breast cancer—six versus twelve months. American Society of Clinical Oncology; 2013.

[19] PHARMAC. New Zealand Pharmaceutical Schedule, August 2011. Wellington: PHARMAC; 2011.

[20] National Institute for Health and Care Excellence. Early and locally advanced breast cancer: diagnosis and treatment. London: National Institute for Health and Care Excellence; 2009.

[21] Liberato NL, Marchetti M, Barosi G. Cost effectiveness of adjuvant trastuzumab in human epidermal growth factor receptor 2–positive breast cancer. J Clin Oncol. 2007;25:625–633. 17308267 10.1200/JCO.2006.06.4220

[22] Lidgren M, Jönsson B, Rehnberg C, Willking N, Bergh J. Cost-effectiveness of HER2 testing and 1-year adjuvant trastuzumab therapy for early breast cancer. Ann Oncol. 2008;19:487–495. 18065409 10.1093/annonc/mdm488

[23] Van Vlaenderen I, Canon J, Cocquyt V, Jerusalem G, Machiels J, Neven P, et al Trastuzumab treatment of early stage breast cancer is cost-effective from the perspective of the Belgian health care authorities. Acta Clin Belg. 2009;64:100–112. 19432022 10.1179/acb.2009.019

[24] Neyt M, Huybrechts M, Hulstaert F, Vrijens F, Ramaekers D. Trastuzumab in early stage breast cancer: a cost-effectiveness analysis for Belgium. Health Policy. 2008;87:146–159. 10.1016/j.healthpol.2007.11.003 18164510

[25] Costilla R, Atkinson J, Blakely T. Incorporating ethnic and deprivation variation to cancer incidence estimates over 2006–2026 for ABC-CBA. Wellington: University of Otago Department of Public Health; 2011.

[26] Dickman PW, Sloggett A, Hills M, Hakulinen T. Regression models for relative survival. Stat Med. 2004;23:51–64. 14695639 10.1002/sim.1597

[27] National Cancer Institute. SEER stat fact sheets: breast cancer. Bethesda (Maryland): National Cancer Institute; 2013.

[28] Blakely T, Costilla R, Soeberg M. Cancer excess mortality rates over 2006–2026 for ABC-CBA. Wellington: University of Otago Department of Public Health; 2012.

[29] Cianfrocca M, Goldstein LJ. Prognostic and predictive factors in early-stage breast cancer. Oncologist. 2004;9:606–616. 15561805 10.1634/theoncologist.9-6-606

[30] Kvizhinadze G, Blakely T. Projected New Zealand lifetables. Wellington: University of Otago Department of Public Health; 2011.

[31] Tan-Chiu E, Yothers G, Romond E, Geyer CE, Ewer M, Keefe D, et al Assessment of cardiac dysfunction in a randomized trial comparing doxorubicin and cyclophosphamide followed by paclitaxel, with or without trastuzumab as adjuvant therapy in node-positive, human epidermal growth factor receptor 2–overexpressing breast cancer: NSABP B-31. J Clin Oncol. 2005;23:7811–7819. 16258083 10.1200/JCO.2005.02.4091

[32] Perez EA, Suman VJ, Davidson NE, Sledge GW, Kaufman PA, Hudis CA, et al Cardiac safety analysis of doxorubicin and cyclophosphamide followed by paclitaxel with or without trastuzumab in the North Central Cancer Treatment Group N9831 adjuvant breast cancer trial. J Clin Oncol. 2008;26:1231–1238. 10.1200/JCO.2007.13.5467 18250349 PMC4048960

[33] Blakely T, Foster R, Wilson N. Burden of Disease Epidemiology, Equity and Cost-Effectiveness (BODE3) study protocol version 2.1. Wellington: University of Otago Department of Public Health; 2012.

[34] Begg S, Vos T, Barker B, Stanley L, Lopez A. Burden of disease and injury in Australia in the new millennium: measuring health loss from diseases, injuries and risk factors. Med J Aust. 2008;188:36–40. 18205562 10.5694/j.1326-5377.2008.tb01503.x

[35] Salomon JA, Vos T, Hogan DR, Gagnon M, Naghavi M, Mokdad A, et al Common values in assessing health outcomes from disease and injury: disability weights measurement study for the Global Burden of Disease Study 2010. Lancet. 2013;380:2129–2143. 10.1016/S0140-6736(12)61680-8 PMC10782811 23245605

[36] Russell SD, Blackwell KL, Lawrence J, Pippen JE, Roe MT, Wood F, et al Independent adjudication of symptomatic heart failure with the use of doxorubicin and cyclophosphamide followed by trastuzumab adjuvant therapy: a combined review of cardiac data from the National Surgical Adjuvant Breast and Bowel Project B-31 and the North Central Cancer Treatment Group N9831 clinical trials. J Clin Oncol. 2010;28:3416–421. 10.1200/JCO.2009.23.6950 20530275

[37] Romond EH, Jeong J-H, Rastogi P, Swain SM, Geyer CE, Ewer MS, et al Seven-year follow-up assessment of cardiac function in NSABP B-31, a randomized trial comparing doxorubicin and cyclophosphamide followed by paclitaxel (ACP) with ACP plus trastuzumab as adjuvant therapy for patients with node-positive, human epidermal growth factor receptor 2–positive breast cancer. J Clin Oncol. 2012;30:3792–3799. 10.1200/JCO.2011.40.0010 22987084 PMC3478574

[38] Foster R, Blakely T, Wilson N, O’Dea D. Protocol for direct costing of health sector interventions for economic modelling (including event pathways). Wellington: University of Otago Department of Public Health; 2013.

[39] Ministry of Health, District Health Boards New Zealand. Purchase Unit Data Dictionary (PUDD) 2011/2012. Wellington: Ministry of Health; 2011.

[40] Ministry of Health. Guide to the National Travel Assistance (NTA) Policy 2005. Wellington: Ministry of Health; 2009.

[41] PHARMAC. Technology assessment report No. 75, with supplementary analysis 75b. Wellington: PHARMAC; 2007.

[42] Ministry of Health, The National Pricing Programme Casemix Cost Weights Project Group. New Zealand casemix framework for publicly funded hospitals (including WIESNZ11 methodology and casemix purchase unit allocation) for the 20011/12 financial year: specification for implementation on NMDS. Wellington: Ministry of Health; 2011.

[43] Ministry of Health. WIESNZ11 cost weights. 2011 [cited 28 Jun 2015]. Available: http://www.health.govt.nz/nz-health-statistics/data-references/weighted-inlier-equivalent-separations/wiesnz11-cost-weights.

[44] van Baal PH, Feenstra TL, Polder JJ, Hoogenveen RT, Brouwer WB. Economic evaluation and the postponement of health care costs. Health Econ. 2011;20:432–445. 10.1002/hec.1599 21210494

[45] Husereau D, Drummond M, Petrou S, Carswell C, Moher D, Greenberg D, et al Consolidated health economic evaluation reporting standards (CHEERS)—explanation and elaboration: a report of the ISPOR health economic evaluation publication guidelines good reporting practices task force. Value Health. 2013;16:231–250. 10.1016/j.jval.2013.02.002 23538175

[46] Sculpher M. Subgroups and heterogeneity in cost-effectiveness analysis. Pharmacoeconomics. 2008;26:799–806. 18767899 10.2165/00019053-200826090-00009

[47] Ramaekers BL, Joore MA, Grutters JP. How should we deal with patient heterogeneity in economic evaluation: a systematic review of national pharmacoeconomic guidelines. Value Health. 2013;16:855–862. 10.1016/j.jval.2013.02.013 23947981

[48] Coyle D, Buxton MJ, O’Brien BJ. Stratified cost‐effectiveness analysis: a framework for establishing efficient limited use criteria. Health Econ. 2003;12:421–427. 12720259 10.1002/hec.788

[49] Grutters JP, Sculpher M, Briggs AH, Severens JL, Candel MJ, Stahl JE, et al Acknowledging patient heterogeneity in economic evaluation. Pharmacoeconomics. 2013;31:111–123. 10.1007/s40273-012-0015-4 23329430

[50] O’Sullivan CC, Bradbury I, Campbell C, Spielmann M, Perez EA, Joensuu H, et al Efficacy of adjuvant trastuzumab for patients with human epidermal growth factor receptor 2–positive early breast cancer and tumors≤ 2 cm: a meta-analysis of the randomized trastuzumab trials. J Clin Oncol. 2015;33:2600–2608. 10.1200/JCO.2015.60.8620 26101239 PMC4534523

[51] Kurian AW, Thompson RN, Gaw AF, Arai S, Ortiz R, Garber AM. A cost-effectiveness analysis of adjuvant trastuzumab regimens in early HER2/neu–positive breast cancer. J Clin Oncol. 2007;25:634–641. 17308268 10.1200/JCO.2006.06.3081

[52] Garrison LP, Lubeck D, Lalla D, Paton V, Dueck A, Perez EA. Cost‐effectiveness analysis of trastuzumab in the adjuvant setting for treatment of HER2‐positive breast cancer. Cancer. 2007;110:489–498. 17592827 10.1002/cncr.22806

[53] PHARMAC. Further supplementary technology assessment report No. 75c. Wellington: PHARMAC; 2008.

[54] Tfayli A, Temraz S, Abou Mrad R, Shamseddine A. Breast cancer in low-and middle-income countries: an emerging and challenging epidemic. J Oncol. 2010;2010:490631 10.1155/2010/490631 21209708 PMC3010663

[55] Zelle SG, Vidaurre T, Abugattas JE, Manrique JE, Sarria G, Jeronimo J, et al Cost-effectiveness analysis of breast cancer control interventions in Peru. PLoS ONE. 2013;8:e82575 10.1371/journal.pone.0082575 24349314 PMC3859673

[56] Bazargani Y, de Boer A, Schellens JH, Leufkens HG, Mantel-Teeuwisse AK. Essential medicines for breast cancer in low and middle income countries. BMC Cancer. 2015;15:591 10.1186/s12885-015-1583-4 26283654 PMC4538762

[57] Dendukuri N, Khetani K, McIsaac M, Brophy J. Testing for HER2-positive breast cancer: a systematic review and cost-effectiveness analysis. CMAJ. 2007;176:1429–1434. 17485695 10.1503/cmaj.061011 PMC1863543

[58] Leopold C, Vogler S, Habl C, Mantel-Teeuwisse AK, Espin J. Personalised medicine as a challenge for public pricing and reimbursement authorities—a survey among 27 European countries on the example of trastuzumab. Health Policy. 2013;113:313–322. 24409503 10.1016/j.healthpol.2013.09.018

[59] Centers for Medicare and Medicaid Services. Current procedural terminology, fourth edition. 2015 [cited 2 Jun 2016]. Available: https://www.cms.gov/apps/ama/license.asp?file=/McrPartBDrugAvgSalesPrice/downloads/Oct_2011_ASP_Pricing_File.zip.

[60] Kresge N, Gokhale K. Roche Herceptin copy’s price still out of reach in India. 2014 Jan 20 [cited 2 Jun 2016]. Bloomberg. Available: http://www.bloomberg.com/news/articles/2014-01-20/roche-herceptin-copy-s-price-still-out-of-reach-in-india.

[61] Nelson KM, Gallagher PC. Biosimilars lining up to compete with Herceptin—opportunity knocks. Expert Opin Ther Pat. 2014;24:1149–1153. 10.1517/13543776.2014.964683 25307085

[62] Hoffmann-La Roche. A study of pertuzumab in addition to chemotherapy and herceptin (trastuzumab) as adjuvant therapy in patients with HER2-positive primary breast cancer. ClinicalTrials.gov NCT01358877. 2011 May 20 [cited 21 Jul 2015]. US National Institutes of Health. Available: https://www.clinicaltrials.gov/ct2/show/NCT01358877.

