# Supplementary figures and images for: Adjuvant Trastuzumab in HER2-Positive Early Breast Cancer by Age and Hormone Receptor Status: A Cost-Utility Analysis
Source: PLoS Med. 2016 Aug 9;13(8):e1002067. doi: 10.1371/journal.pmed.1002067 (PMC4978494; doi:10.1371/journal.pmed.1002067)

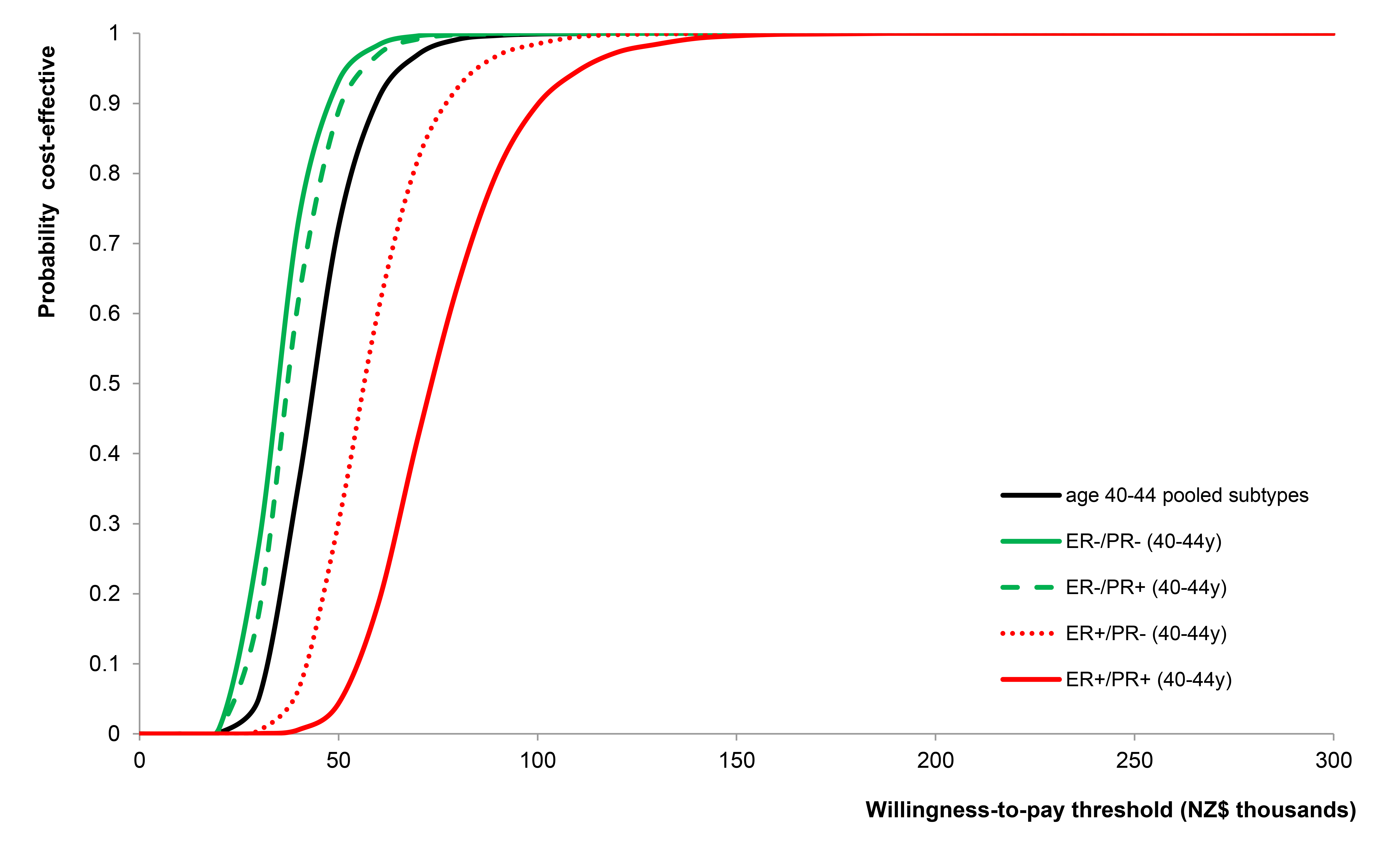

Supplement: S1 Fig — (TIF) [file pmed.1002067.s001.tif]

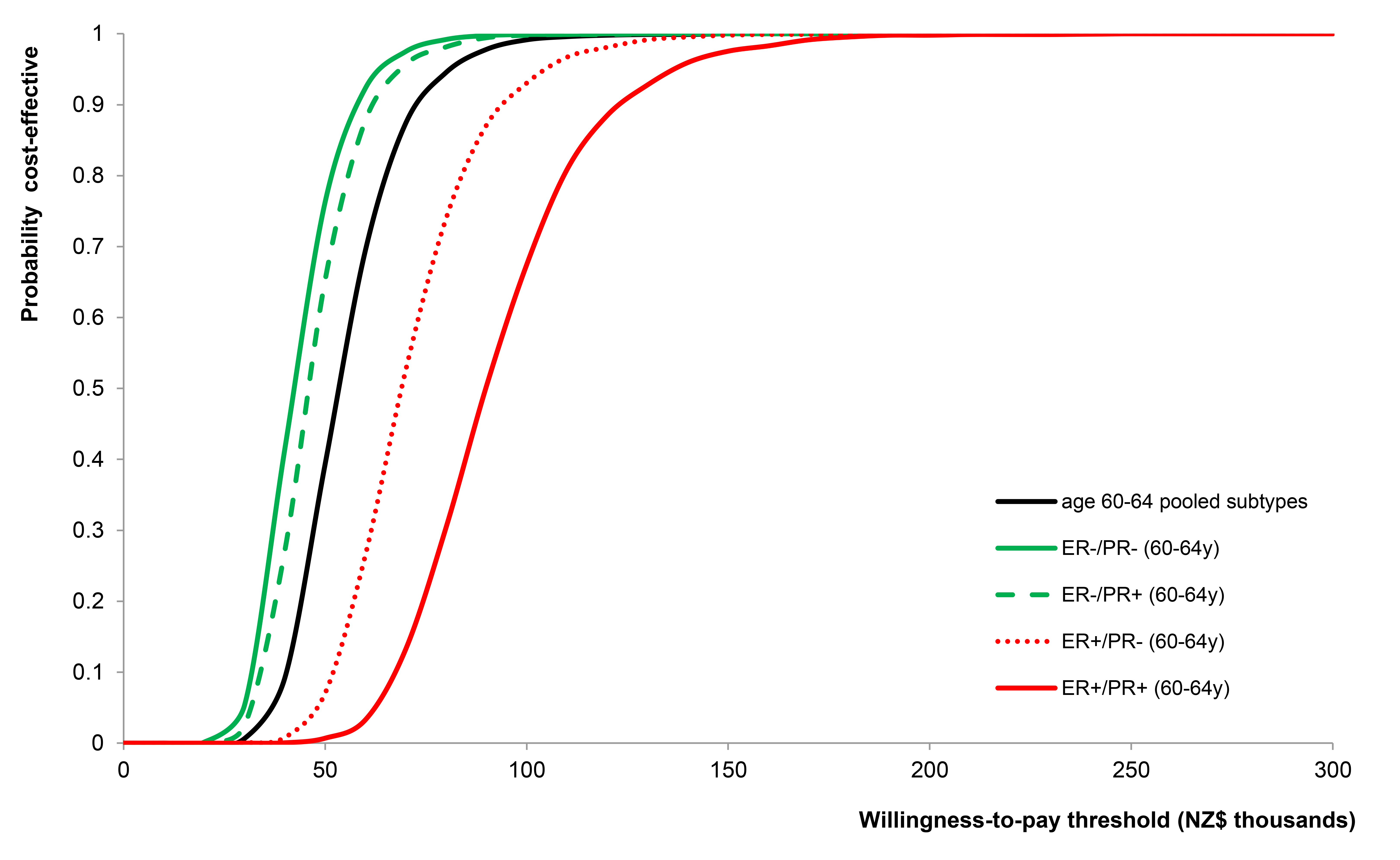

Supplement: S2 Fig — (TIF) [file pmed.1002067.s002.tif]

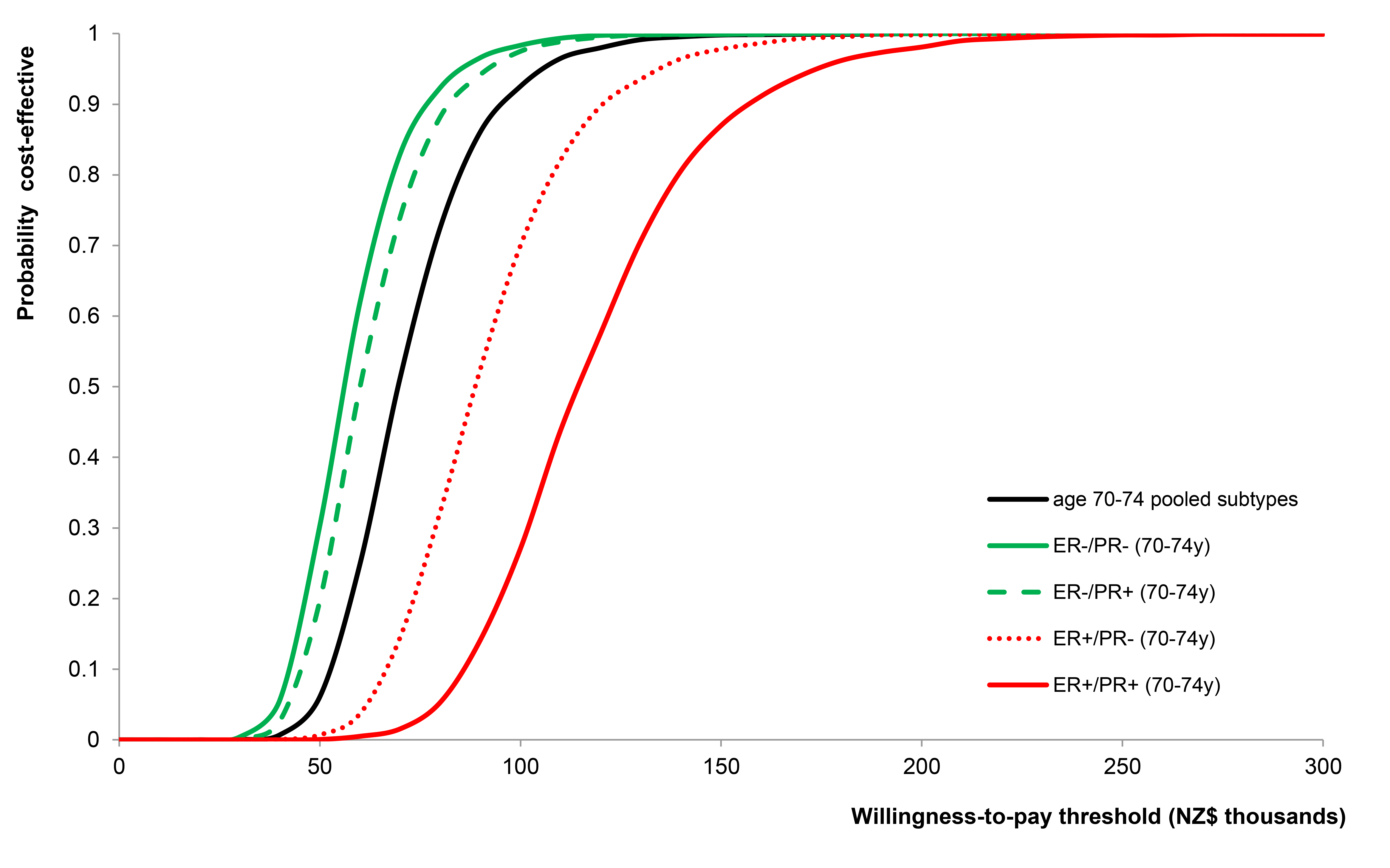

Supplement: S3 Fig — (TIF) [file pmed.1002067.s003.tif]

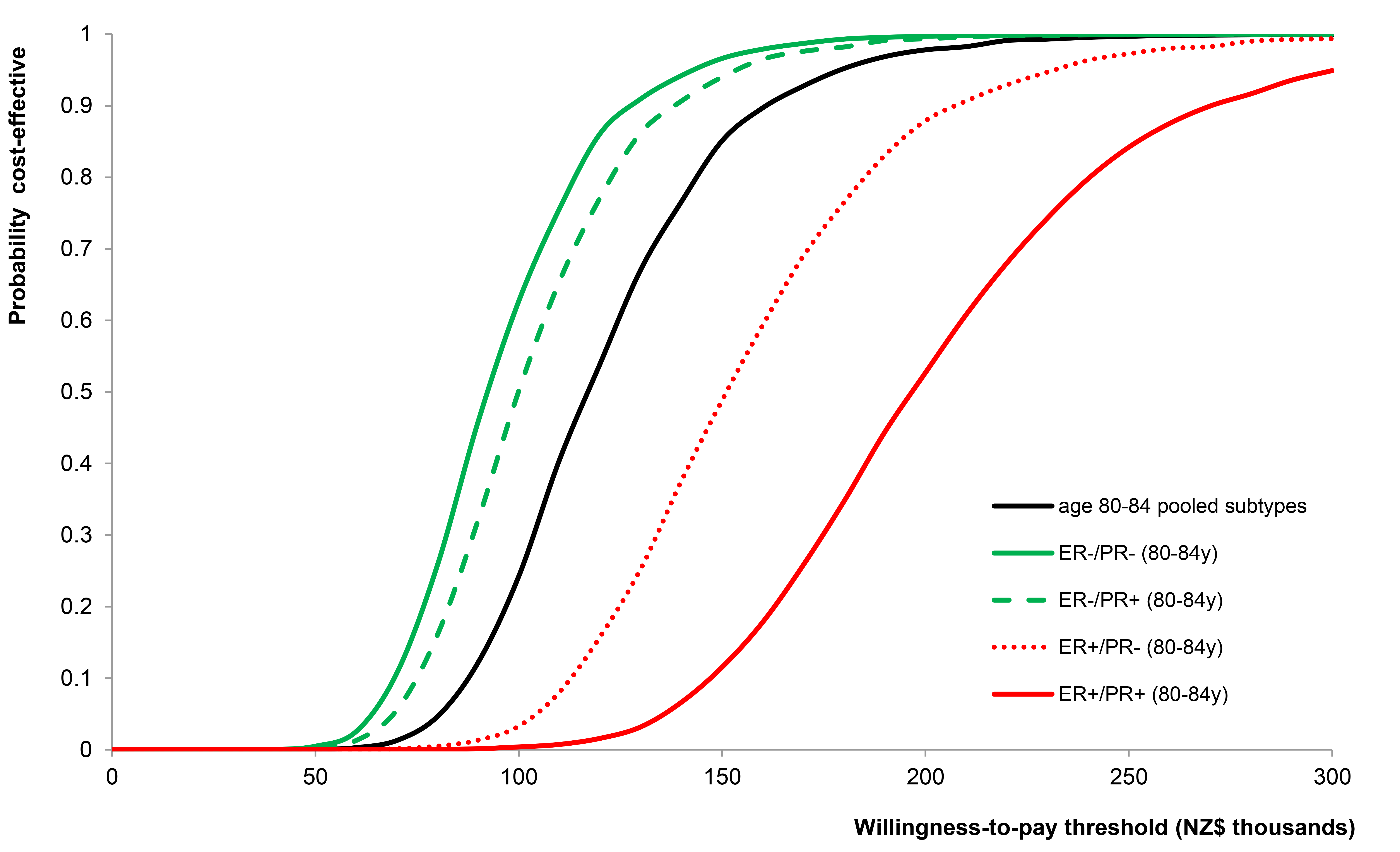

Supplement: S4 Fig — (TIF) [file pmed.1002067.s004.tif]
