# Supplementary material for: Adjuvant Trastuzumab in HER2-Positive Early Breast Cancer by Age and Hormone Receptor Status: A Cost-Utility Analysis
Source: PLoS Med. 2016 Aug 9;13(8):e1002067. doi: 10.1371/journal.pmed.1002067 (PMC4978494; doi:10.1371/journal.pmed.1002067)
Supplement: S1 Table — (DOCX) [file pmed.1002067.s006.docx]

| Age Group | ER+/PR+/ HER2+ | ER+/PR-/ HER2+ | ER-/PR+/ HER2+ | ER-/PR-/ HER2+ |
| --- | --- | --- | --- | --- |
| 25-29 y | 79.6% | 72.6% | 55.2% | 50.4% |
| 30-34 y | 79.8% | 72.8% | 55.5% | 50.6% |
| 35-39 y | 79.9% | 73.0% | 55.8% | 50.9% |
| 40-44 y | 78.9% | 71.7% | 54.0% | 49.0% |
| 45-49 y | 82.9% | 76.9% | 61.3% | 56.8% |
| 50-54 y | 83.0% | 77.0% | 61.5% | 57.0% |
| 55-59 y | 81.5% | 75.0% | 58.6% | 53.9% |
| 60-64 y | 77.8% | 70.3% | 51.9% | 46.9% |
| 65-69 y | 73.0% | 64.3% | 44.0% | 38.7% |
| 70-74 y | 73.0% | 64.3% | 44.0% | 38.7% |
| 75-79 y | 71.2% | 62.1% | 41.2% | 35.9% |
| 80-84 y | 70.2% | 60.9% | 39.7% | 34.4% |
| 85-89 y | 70.3% | 61.0% | 39.9% | 34.5% |
| 90-94 y | 70.4% | 61.1% | 40.0% | 34.6% |
| *ER* estrogen receptor; *HER2* human epidermal growth factor receptor 2;  *PR* progesterone receptor. | | | | |
