# Supplementary material for: Adjuvant Trastuzumab in HER2-Positive Early Breast Cancer by Age and Hormone Receptor Status: A Cost-Utility Analysis
Source: PLoS Med. 2016 Aug 9;13(8):e1002067. doi: 10.1371/journal.pmed.1002067 (PMC4978494; doi:10.1371/journal.pmed.1002067)
Supplement: S2 Table — (DOCX) [file pmed.1002067.s007.docx]

| **Breast cancer subtypes** | **ER+/PR+/ HER2+** | **ER+/PR-/ HER2+** | **ER-/PR+/ HER2+** | **ER-/PR-/ HER2+** | ER+/PR+/ HER2- | ER+/PR-/ HER2- | ER-/PR+/ HER2- | ER-/PR-/ HER2- |
| --- | --- | --- | --- | --- | --- | --- | --- | --- |
| EMRR (Parise et al)* | 0.3349 | 0.4703 | 0.8766 | 1.015 | 0.1349 | 0.3108 | 0.6988 | 1 |
| **Age < 40 y** |  |  |  |  |  |  |  |  |
| NZ proportion | 0.1940 | 0.0448 | 0.0119 | 0.1228 | 0.4036 | 0.0512 | 0.0158 | 0.1558 |
| Floating EMRR | 0.7307 | 1.0262 | 1.9126 | 2.2136 | 0.2943 | 0.6781 | 1.5248 | 2.1820 |
| Intercept adjustment | -0.3138 | 0.0259 | 0.6485 | 0.7946 | -1.2231 | -0.3885 | 0.4219 | 0.7802 |
| **Age 40 – 59 y** |  |  |  |  |  |  |  |  |
| NZ proportion | 0.1249 | 0.0289 | 0.0076 | 0.0791 | 0.4893 | 0.0621 | 0.0192 | 0.1889 |
| Floating EMRR | 0.7788 | 1.0938 | 2.0387 | 2.3595 | 0.3137 | 0.7228 | 1.6254 | 2.3258 |
| Intercept adjustment | -0.2500 | 0.0897 | 0.7123 | 0.8585 | -1.1592 | -0.3247 | 0.4857 | 0.8441 |
| **Age 60 – 79 y** |  |  |  |  |  |  |  |  |
| NZ proportion | 0.0952 | 0.0330 | 0.0042 | 0.0596 | 0.5682 | 0.1126 | 0.0080 | 0.1193 |
| Floating EMRR | 0.9622 | 1.3514 | 2.5187 | 2.9151 | 0.3876 | 0.8929 | 2.0080 | 2.8734 |
| Intercept adjustment | -0.0385 | 0.3011 | 0.9237 | 1.0699 | -0.9478 | -0.1132 | 0.6971 | 1.0555 |
| **Age ≥ 80 y** |  |  |  |  |  |  |  |  |
| NZ proportion | 0.0668 | 0.0231 | 0.0030 | 0.0418 | 0.6086 | 0.1206 | 0.0085 | 0.1278 |
| Floating EMRR | 1.0102 | 1.4188 | 2.6444 | 3.0606 | 0.4069 | 0.9375 | 2.1083 | 3.0168 |
| Intercept adjustment | 0.0102 | 0.3498 | 0.9725 | 1.1186 | -0.8991 | -0.0645 | 0.7459 | 1.1042 |
| *EMR* excess mortality rate; *EMRR* excess mortality rate ratio; *ER* estrogen receptor; *HER2* human epidermal growth factor receptor 2; *NZ* New Zealand;  *PR* progesterone receptor. * Reference [3] in manuscript. | | | | | | | | |
