# Supplementary material for: Adjuvant Trastuzumab in HER2-Positive Early Breast Cancer by Age and Hormone Receptor Status: A Cost-Utility Analysis
Source: PLoS Med. 2016 Aug 9;13(8):e1002067. doi: 10.1371/journal.pmed.1002067 (PMC4978494; doi:10.1371/journal.pmed.1002067)
Supplement: S3 Table — (DOCX) [file pmed.1002067.s008.docx]

| **Age Group** | **HER2+ subtype** | *ER+/PR+* | *ER+/PR–* | *ER–/PR+* | *ER–/PR–* | *Pooled* |
| --- | --- | --- | --- | --- | --- | --- |
| 25-29 y | Incr. QALYs | 1.09 | 1.43 | 2.16 | 2.33 | 1.84 |
|  | Incr. costs | 70,925 | 70,661 | 69,757 | 69,422 | 70,206 |
|  | ICER | *64,932* | *49,440* | *32,323* | *29,830* | *38,181* |
| 30-34 y | Incr. QALYs | 1.03 | 1.35 | 2.04 | 2.20 | 1.73 |
|  | Incr. costs | 71,268 | 71,117 | 70,468 | 70,196 | 70,805 |
|  | ICER | *69,362* | *52,848* | *34,583* | *31,917* | *40,839* |
| 35-39 y | Incr. QALYs | 0.96 | 1.26 | 1.91 | 2.06 | 1.62 |
|  | Incr. costs | 71,597 | 71,559 | 71,167 | 70,960 | 71,389 |
|  | ICER | *74,797* | *57,013* | *37,315* | *34,430* | *44,068* |
| 40-44 y | Incr. QALYs | 0.97 | 1.27 | 1.91 | 2.06 | 1.63 |
|  | Incr. costs | 72,004 | 72,093 | 71,959 | 71,806 | 72,067 |
|  | ICER | *74,288* | *56,867* | *37,626* | *34,827* | *44,269* |
| 45-49 y | Incr. QALYs | 0.73 | 0.97 | 1.52 | 1.67 | 1.27 |
|  | Incr. costs | 72,143 | 72,339 | 72,597 | 72,590 | 72,505 |
|  | ICER | *99,282* | *74,932* | *47,620* | *43,502* | *57,123* |
| 50-54 y | Incr. QALYs | 0.74 | 0.99 | 1.57 | 1.72 | 1.31 |
|  | Incr. costs | 72,581 | 72,951 | 73,652 | 73,771 | 73,379 |
|  | ICER | *98,134* | *74,039* | *46,949* | *42,843* | *56,050* |
| 55-59 y | Incr. QALYs | 0.70 | 0.93 | 1.46 | 1.60 | 1.22 |
|  | Incr. costs | 72,733 | 73,172 | 73,993 | 74,130 | 72,393 |
|  | ICER | *103,859* | *78,745* | *50,598* | *46,361* | *59,391* |
| 60-64 y | Incr. QALYs | 0.81 | 1.06 | 1.63 | 1.77 | 1.37 |
|  | Incr. costs | 73,336 | 73,981 | 75,161 | 75,353 | 74,648 |
|  | ICER | *90,571* | *69,472* | *46,006* | *42,534* | *54,316* |
| 65-69 y | Incr. QALYs | 0.81 | 1.05 | 1.54 | 1.65 | 1.32 |
|  | Incr. costs | 73,326 | 73,971 | 74,946 | 75,021 | 74,556 |
|  | ICER | *90,985* | *70,760* | *48,622* | *45,468* | *56,419* |
| 70-74 y | Incr. QALYs | 0.63 | 0.82 | 1.22 | 1.31 | 1.04 |
|  | Incr. costs | 72,426 | 72,959 | 73,765 | 73,825 | 73,440 |
|  | ICER | *115,456* | *89,363* | *60,661* | *56,522* | *70,792* |
| 75-79 y | Incr. QALYs | 0.48 | 0.63 | 0.93 | 1.00 | 0.80 |
|  | Incr. costs | 70,347 | 70,423 | 70,035 | 69,738 | 70,273 |
|  | ICER | *145,119* | *111,891* | *75,196* | *69,854* | *88,169* |
| 80-84 y | Incr. QALYs | 0.34 | 0.45 | 0.66 | 0.71 | 0.57 |
|  | Incr. costs | 68,066 | 67,915 | 67,071 | 66,679 | 67,509 |
|  | ICER | *198,669* | *152,339* | *100,931* | *93,369* | *119,142* |
| 85-89 y | Incr. QALYs | 0.22 | 0.28 | 0.43 | 0.47 | 0.37 |
|  | Incr. costs | 64,376 | 64,031 | 62,823 | 62,366 | 63,418 |
|  | ICER | *296,081* | *224,778* | *144,770* | *132,745* | *173,219* |
| 90-94 y | Incr. QALYs | 0.13 | 0.17 | 0.27 | 0.29 | 0.22 |
|  | Incr. costs | 58,698 | 58,241 | 56,826 | 56,335 | 57,511 |
|  | ICER | *452,541* | *340,275* | *212,759* | *193,195* | *258,261* |
| *ER* estrogen receptor; *HER2* human epidermal growth factor receptor 2; *ICER* incremental cost-effectiveness ratio; *PR* progesterone receptor; QALY quality-adjusted life-year. | | | | | | |
