# Supplementary material for: Adjuvant Trastuzumab in HER2-Positive Early Breast Cancer by Age and Hormone Receptor Status: A Cost-Utility Analysis
Source: PLoS Med. 2016 Aug 9;13(8):e1002067. doi: 10.1371/journal.pmed.1002067 (PMC4978494; doi:10.1371/journal.pmed.1002067)
Supplement: S4 Table — (DOCX) [file pmed.1002067.s009.docx]

| **Age Group** | **HER2+ subtype** | *ER+/PR+* | *ER+/PR–* | *ER–/PR+* | *ER–/PR–* | *Pooled* |
| --- | --- | --- | --- | --- | --- | --- |
| 25-29 y | Incr. QALYs | 1.00 | 1.32 | 2.05 | 2.23 | 1.64 |
|  | Incr. costs | 71,117 | 70,927 | 70,233 | 69,964 | *70,543* |
|  | ICER | *70,919* | *53,559* | *34,197* | *31,315* | *43,102* |
| 30-34 y | Incr. QALYs | 0.95 | 1.26 | 1.95 | 2.12 | 1.55 |
|  | Incr. costs | 71,437 | 71,353 | 70,903 | 70,697 | *71,076* |
|  | ICER | *75,127* | *56,796* | *36,345* | *33,299* | *45,731* |
| 35-39 y | Incr. QALYs | 0.89 | 1.18 | 1.83 | 2.00 | 1.46 |
|  | Incr. costs | 71,748 | 71,770 | 71,572 | 71,431 | *71,603* |
|  | ICER | *80,468* | *60,877* | *39,006* | *35,743* | *49,006* |
| 40-44 y | Incr. QALYs | 0.91 | 1.20 | 1.85 | 2.01 | 1.48 |
|  | Incr. costs | 72,143 | 72,291 | 72,358 | 72,276 | *72,232* |
|  | ICER | *79,316* | *60,264* | *39,045* | *35,897* | *48,867* |
| 45-49 y | Incr. QALYs | 0.68 | 0.91 | 1.47 | 1.62 | 1.16 |
|  | Incr. costs | 72,253 | 72,488 | 72,890 | 72,939 | *72,616* |
|  | ICER | *105,792* | *79,383* | *49,632* | *45,103* | *62,521* |
| 50-54 y | Incr. QALYs | 0.70 | 0.94 | 1.52 | 1.67 | 1.20 |
|  | Incr. costs | 72,688 | 73,092 | 73,930 | 74,104 | *73,423* |
|  | ICER | *104,065* | *78,090* | *48,777* | *44,298* | *61,335* |
| 55-59 y | Incr. QALYs | 0.67 | 0.89 | 1.42 | 1.56 | 1.13 |
|  | Incr. costs | 72,860 | 73,332 | 74,296 | 74,491 | *73,708* |
|  | ICER | *109,520* | *82,578* | *52,255* | *47,648* | *65,487* |
| 60-64 y | Incr. QALYs | 0.77 | 1.02 | 1.60 | 1.74 | 1.27 |
|  | Incr. costs | 73,479 | 74,161 | 75,518 | 75,783 | *74,679* |
|  | ICER | *95,112* | *72,507* | *47,223* | *43,434* | *58,692* |
| 65-69 y | Incr. QALYs | 0.77 | 1.01 | 1.52 | 1.64 | 1.22 |
|  | Incr. costs | 73,512 | 74,205 | 75,405 | 75,571 | *74,599* |
|  | ICER | *95,254* | *73,550* | *49,579* | *46,091* | *61,130* |
| 70-74 y | Incr. QALYs | 0.60 | 0.79 | 1.20 | 1.29 | 0.96 |
|  | Incr. costs | 72,664 | 73,232 | 74,223 | 74,361 | *73,559* |
|  | ICER | *121,320* | *93,238* | *62,103* | *57,530* | *76,871* |
| 75-79 y | Incr. QALYs | 0.46 | 0.60 | 0.91 | 0.98 | 0.73 |
|  | Incr. costs | 70,685 | 70,813 | 70,642 | 70,432 | *70,589* |
|  | ICER | *153,830* | *117,791* | *77,711* | *71,783* | *96,831* |
| 80-84 y | Incr. QALYs | 0.32 | 0.42 | 0.64 | 0.69 | 0.51 |
|  | Incr. costs | 68,331 | 68,221 | 67,550 | 67,227 | *67,794* |
|  | ICER | *213,827* | *162,858* | *105,938* | *97,448* | *132,762* |
| 85-89 y | Incr. QALYs | 0.20 | 0.26 | 0.41 | 0.44 | 0.33 |
|  | Incr. costs | 64,410 | 64,105 | 63,044 | 62,643 | *63,528* |
|  | ICER | *322,696* | *243,574* | *154,357* | *140,814* | *195,009* |
| 90-94 y | Incr. QALYs | 0.12 | 0.16 | 0.25 | 0.27 | 0.20 |
|  | Incr. costs | 58,724 | 58,312 | 57,039 | 56,596 | *57,654* |
|  | ICER | *494,304* | *369,987* | *228,280* | *206,404* | *290,472* |
| *ER* estrogen receptor; *HER2* human epidermal growth factor receptor 2; *ICER* incremental cost-effectiveness ratio; *PR* progesterone receptor; QALY quality-adjusted life-year. | | | | | | |
