# Supplementary material for: Adjuvant Trastuzumab in HER2-Positive Early Breast Cancer by Age and Hormone Receptor Status: A Cost-Utility Analysis
Source: PLoS Med. 2016 Aug 9;13(8):e1002067. doi: 10.1371/journal.pmed.1002067 (PMC4978494; doi:10.1371/journal.pmed.1002067)
Supplement: S5 Table — (DOCX) [file pmed.1002067.s010.docx]

| **Age Group** | **HER2+ subtype** | *ER+/PR+* | *ER+/PR–* | *ER–/PR+* | *ER–/PR–* | *Pooled* |
| --- | --- | --- | --- | --- | --- | --- |
| 25-29 y | Incr. QALYs | 1.13 | 1.47 | 2.18 | 2.34 | 1.76 |
|  | Incr. costs | 70,754 | 70,431 | 69,377 | 68,999 | *69,874* |
|  | ICER | *62,820* | *48,027* | *31,782* | *29,452* | *39,782* |
| 30-34 y | Incr. QALYs | 1.06 | 1.39 | 2.06 | 2.21 | 1.66 |
|  | Incr. costs | 71,064 | 70,844 | 70,008 | 69,682 | *70,377* |
|  | ICER | *66,793* | *51,146* | *33,967* | *31,503* | *42,431* |
| 35-39 y | Incr. QALYs | 0.99 | 1.29 | 1.93 | 2.07 | 1.55 |
|  | Incr. costs | 71,370 | 71,254 | 70,647 | 70,374 | *70,883* |
|  | ICER | *71,928* | *55,137* | *36,691* | *34,041* | *45,759* |
| 40-44 y | Incr. QALYs | 1.00 | 1.30 | 1.92 | 2.05 | 1.55 |
|  | Incr. costs | 71,751 | 71,754 | 71,374 | 71,145 | *71,467* |
|  | ICER | *71,814* | *55,305* | *37,238* | *34,667* | *46,248* |
| 45-49 y | Incr. QALYs | 0.75 | 0.99 | 1.53 | 1.67 | 1.22 |
|  | Incr. costs | 71,914 | 72,041 | 72,094 | 72,020 | *71,986* |
|  | ICER | *96,392* | *73,090* | *47,080* | *43,200* | *59,003* |
| 50-54 y | Incr. QALYs | 0.75 | 1.00 | 1.57 | 1.71 | 1.24 |
|  | Incr. costs | 72,312 | 72,613 | 73,098 | 73,144 | *72,755* |
|  | ICER | *96,014* | *72,744* | *46,684* | *42,768* | *58,439* |
| 55-59 y | Incr. QALYs | 0.71 | 0.93 | 1.45 | 1.57 | 1.15 |
|  | Incr. costs | 72,408 | 72,774 | 73,371 | 73,432 | *72,953* |
|  | ICER | *102,503* | *78,041* | *50,741* | *46,671* | *63,291* |
| 60-64 y | Incr. QALYs | 0.81 | 1.06 | 1.60 | 1.73 | 1.28 |
|  | Incr. costs | 72,942 | 73,504 | 74,432 | 74,539 | *73,790* |
|  | ICER | *90,056* | *69,357* | *46,450* | *43,100* | *57,425* |
| 65-69 y | Incr. QALYs | 0.80 | 1.03 | 1.50 | 1.60 | 1.22 |
|  | Incr. costs | 72,880 | 73,442 | 74,162 | 74,154 | *73,576* |
|  | ICER | *90,905* | *70,990* | *49,337* | *46,303* | *60,428* |
| 70-74 y | Incr. QALYs | 0.62 | 0.81 | 1.19 | 1.27 | 0.96 |
|  | Incr. costs | 71,973 | 72,444 | 73,058 | 73,053 | *72,562* |
|  | ICER | *115,405* | *89,644* | *61,449* | *57,431* | *75,582* |
| 75-79 y | Incr. QALYs | 0.48 | 0.63 | 0.92 | 0.98 | 0.74 |
|  | Incr. costs | 69,878 | 69,904 | 69,349 | 68,994 | *69,469* |
|  | ICER | *144,175* | *111,504* | *75,553* | *70,364* | *93,605* |
| 80-84 y | Incr. QALYs | 0.35 | 0.45 | 0.67 | 0.71 | 0.54 |
|  | Incr. costs | 67,746 | 67,569 | 66,631 | 66,204 | *66,992* |
|  | ICER | *195,038* | *149,952* | *100,061* | *92,769* | *124,700* |
| 85-89 y | Incr. QALYs | 0.22 | 0.29 | 0.44 | 0.48 | 0.35 |
|  | Incr. costs | 64,356 | 63,995 | 62,730 | 62,251 | *63,306* |
|  | ICER | *287,895* | *218,995* | *141,830* | *130,278* | *178,409* |
| 90-94 y | Incr. QALYs | 0.13 | 0.18 | 0.27 | 0.30 | 0.22 |
|  | Incr. costs | 58,715 | 58,242 | 56,774 | 56,264 | *57,483* |
|  | ICER | *439,398* | *330,892* | *207,811* | *188,973* | *263,657* |
| *ER* estrogen receptor; *HER2* human epidermal growth factor receptor 2; *ICER* incremental cost-effectiveness ratio; *PR* progesterone receptor; QALY quality-adjusted life-year. | | | | | | |
