# Supplementary material for: Adjuvant Trastuzumab in HER2-Positive Early Breast Cancer by Age and Hormone Receptor Status: A Cost-Utility Analysis
Source: PLoS Med. 2016 Aug 9;13(8):e1002067. doi: 10.1371/journal.pmed.1002067 (PMC4978494; doi:10.1371/journal.pmed.1002067)
Supplement: S6 Table — (DOCX) [file pmed.1002067.s011.docx]

| **Incr. QALYs** | *ER+/PR+* | *ER+/PR–* | *ER–/PR+* | *ER–/PR–* | *Pooled* |
| --- | --- | --- | --- | --- | --- |
| 25-44 y | 1.38 | 1.81 | 2.78 | 2.09 | 1.80 |
| 45-54 y | 1.03 | 1.38 | 2.22 | 1.70 | 1.42 |
| 55-64 y | 1.08 | 1.43 | 2.26 | 1.69 | 1.44 |
| 65-74 y | 1.03 | 1.35 | 2.05 | 1.49 | 1.32 |
| 75-84 y | 0.59 | 0.78 | 1.19 | 0.87 | 0.76 |
| ≥ 85 y | 0.27 | 0.36 | 0.57 | 0.42 | 0.36 |
| **Incr. costs** | *ER+/PR+* | *ER+/PR–* | *ER–/PR+* | *ER–/PR–* | *Pooled* |
| 25-44 y | 72,575 | 72,861 | 73,236 | 71,304 | 72,066 |
| 45-54 y | 73,267 | 73,872 | 75,175 | 73,202 | 73,386 |
| 55-64 y | 74,380 | 75,395 | 77,597 | 74,791 | 74,814 |
| 65-74 y | 74,535 | 75,687 | 77,976 | 74,469 | 74,782 |
| 75-84 y | 70,463 | 70,850 | 71,316 | 68,307 | 69,576 |
| ≥ 85 y | 63,424 | 63,257 | 62,555 | 60,753 | 62,171 |
| **ICERs** | *ER+/PR+* | *ER+/PR–* | *ER–/PR+* | *ER–/PR–* | *Pooled* |
| 25-44 y | 52,799 | 40,277 | 26,341 | 34,206 | 39,964 |
| 45-54 y | 70,862 | 53,452 | 33,815 | 43,160 | 51,682 |
| 55-64 y | 69,476 | 52,964 | 34,399 | 44,293 | 51,889 |
| 65-74 y | 73,500 | 56,908 | 38,463 | 50,564 | 56,830 |
| 75-84 y | 121,774 | 93,300 | 61,455 | 80,857 | 91,322 |
| ≥ 85 y | 241,179 | 181,561 | 114,036 | 148,919 | 171,281 |
| *ER* estrogen receptor; *ICER* incremental cost-effectiveness ratio; *PR* progesterone receptor; *QALY^DW^* quality-adjusted life-year (disability weights). | | | | | |
