# Supplementary material for: Adjuvant Trastuzumab in HER2-Positive Early Breast Cancer by Age and Hormone Receptor Status: A Cost-Utility Analysis
Source: PLoS Med. 2016 Aug 9;13(8):e1002067. doi: 10.1371/journal.pmed.1002067 (PMC4978494; doi:10.1371/journal.pmed.1002067)
Supplement: S7 Table — (DOCX) [file pmed.1002067.s012.docx]

| **Age Group** | **HER2+ subtype** | *ER+/PR+* | *ER+/PR–* | *ER–/PR+* | *ER–/PR–* | *Pooled* |
| --- | --- | --- | --- | --- | --- | --- |
| 25-29 y | Incr. QALYs | 1.09 | 1.43 | 2.16 | 2.33 | 1.84 |
|  | Incr. costs | 51,464 | 51,277 | 50,603 | 50,345 | 50,949 |
|  | ICER | *47,115* | *35,878* | *23,448* | *21,633* | *27,708* |
| 30-34 y | Incr. QALYs | 1.03 | 1.35 | 2.04 | 2.20 | 1.73 |
|  | Incr. costs | 51,809 | 51,735 | 51,314 | 51,119 | 51,548 |
|  | ICER | *50,423* | *38,445* | *25,183* | *23,243* | *29,732* |
| 35-39 y | Incr. QALYs | 0.96 | 1.26 | 1.91 | 2.06 | 1.62 |
|  | Incr. costs | 52,142 | 52,179 | 52,014 | 51,883 | 52,134 |
|  | ICER | *54,472* | *41,572* | *27,273* | *25,174* | *32,182* |
| 40-44 y | Incr. QALYs | 0.97 | 1.27 | 1.91 | 2.06 | 1.63 |
|  | Incr. costs | 52,564 | 52,732 | 52,835 | 52,762 | 52,836 |
|  | ICER | *54,231* | *41,595* | *27,627* | *25,590* | *32,456* |
| 45-49 y | Incr. QALYs | 0.73 | 0.97 | 1.52 | 1.67 | 1.27 |
|  | Incr. costs | 52,672 | 52,931 | 53,376 | 53,433 | 53,201 |
|  | ICER | *72,486* | *54,828* | *35,013* | *32,022* | *41,915* |
| 50-54 y | Incr. QALYs | 0.74 | 0.99 | 1.57 | 1.72 | 1.31 |
|  | Incr. costs | 53,121 | 53,552 | 54,434 | 54,614 | 54,081 |
|  | ICER | *71,823* | *54,351* | *34,698* | *31,717* | *41,309* |
| 55-59 y | Incr. QALYs | 0.70 | 0.93 | 1.46 | 1.60 | 1.22 |
|  | Incr. costs | 53,315 | 53,820 | 54,839 | 55,043 | 53,265 |
|  | ICER | *76,131* | *57,920* | *37,500* | *34,424* | *43,780* |
| 60-64 y | Incr. QALYs | 0.81 | 1.06 | 1.63 | 1.77 | 1.37 |
|  | Incr. costs | 53,990 | 54,713 | 56,125 | 56,395 | 55,508 |
|  | ICER | *66,679* | *51,378* | *34,354* | *31,833* | *40,389* |
| 65-69 y | Incr. QALYs | 0.81 | 1.05 | 1.54 | 1.65 | 1.32 |
|  | Incr. costs | 54,108 | 54,854 | 56,129 | 56,305 | 55,603 |
|  | ICER | *67,139* | *52,473* | *36,415* | *34,125* | *42,077* |
| 70-74 y | Incr. QALYs | 0.63 | 0.82 | 1.22 | 1.31 | 1.04 |
|  | Incr. costs | 53,330 | 53,962 | 55,067 | 55,228 | 54,606 |
|  | ICER | *85,014* | *66,096* | *45,285* | *42,284* | *52,637* |
| 75-79 y | Incr. QALYs | 0.48 | 0.63 | 0.93 | 1.00 | 0.80 |
|  | Incr. costs | 51,679 | 51,939 | 52,088 | 51,970 | 52,075 |
|  | ICER | *106,609* | *82,522* | *55,927* | *52,057* | *65,336* |
| 80-84 y | Incr. QALYs | 0.34 | 0.45 | 0.66 | 0.71 | 0.57 |
|  | Incr. costs | 49,820 | 49,855 | 49,558 | 49,348 | 49,740 |
|  | ICER | *145,413* | *111,830* | *74,577* | *69,101* | *87,783* |
| 85-89 y | Incr. QALYs | 0.22 | 0.28 | 0.43 | 0.47 | 0.37 |
|  | Incr. costs | 46,933 | 46,765 | 46,077 | 45,793 | 46,428 |
|  | ICER | *215,855* | *164,165* | *106,179* | *97,468* | *126,813* |
| 90-94 y | Incr. QALYs | 0.13 | 0.17 | 0.27 | 0.29 | 0.22 |
|  | Incr. costs | 42,672 | 42,378 | 41,439 | 41,106 | 41,901 |
|  | ICER | *328,991* | *247,593* | *155,150* | *140,970* | *188,164* |
| *ER* estrogen receptor; *HER2* human epidermal growth factor receptor 2; *ICER* incremental cost-effectiveness ratio; *PR* progesterone receptor; QALY quality-adjusted life-year. | | | | | | |
