# Supplementary material for: Adjuvant Trastuzumab in HER2-Positive Early Breast Cancer by Age and Hormone Receptor Status: A Cost-Utility Analysis
Source: PLoS Med. 2016 Aug 9;13(8):e1002067. doi: 10.1371/journal.pmed.1002067 (PMC4978494; doi:10.1371/journal.pmed.1002067)
Supplement: S1 Text — (DOCX) [file pmed.1002067.s013.docx]

**S1 Text (Supporting Information) for**

**“Adjuvant Trastuzumab in HER2-Positive Early Breast Cancer by Age and Hormone Receptor Status: A Cost-Utility Analysis”**

**EMR & HR calibration**

This supporting information text (S1 Text) provides further details of the excess mortality rate (EMR) and hazard ratio (HR) calibration in the *Methods* of the main manuscript. Breast cancer mortality rates were estimated using excess mortality rate (EMR) (the ‘extra’ mortality from breast cancer, the mortality analogue of relative survival) modelling [1] on New Zealand Cancer Registry data linked to mortality data. We had excess mortality rate equations by SEER cancer stage (ie local and regional) [2] from previous work [3]. However, EMRs by local or regional stage do not capture the heterogeneity in prognosis by other clinical characteristics. Prognosis even within regional breast cancer varies markedly by estrogen receptor (ER) progesterone receptor (PR) status, human epidermal growth factor receptor 2 (HER2) status, tumor grade, etc [4]. In order to incorporate this heterogeneity by prognosis into our analysis, we recalibrated the existing EMR equations to match four more detailed invasive early breast cancer subtypes, as defined by regional spread, ER status and PR status. The four subtypes reflect the spectrum of prognosis in HER2+ early invasive breast cancer, from best prognosis to worst prognosis. All four subtypes and their five-year relative survival ratios (RSRs) are provided in S1 Table. The RSRs for these subtypes were not available from the outset; they were retrospectively calculated from the EMRs once these were available at the end of the process described below.

To reiterate, what we had from earlier work were existing EMR equations by SEER stage (local and regional) and time since diagnosis. What we needed were EMRs for each of the four breast cancer subtypes above. Thus we needed to ascertain by how much to recalibrate the existing EMR equations (ie by how much we needed to move the intercept on the y-axis up or down). To do this we used a six-step process explained below.

Population-based data from the United States (Parise et al) [5] were used to calculate an EMR ratio (EMRR) for each of the eight possible combination of ER status/PR status/HER2 status, using ER-/PR-/HER2- as the reference category (workings available on request).

1. A ‘floating’ EMRR for each combination of ER status/PR status/HER2 status by 20-year-wide age-bands was created. The relative proportions of the eight subtypes from step *i*, above, was only available for two age groups (age <50 years; age ≥50 years). The Parise et al proportions of HER2+ prevalence by the two age groups closely matched those of the NZ Cancer Registry’s 2001-05 HER2+ prevalence by age (provided in 5-year-wide age-bands); the absolute difference was -0.13%, in those age <50 years, and 0.26%, in those age ≥50 years. The Parise et al HER2+ proportions for the ER/PR subtypes by age group were applied to NZ HER2+ prevalence by age. These counts were used to estimate the NZ proportions of the eight subtypes by age-bands. EMRRs from step *i* were adjusted using these NZ proportions to create ‘floating’ EMRRs (workings available on request).
2. The intercept adjustment was calculated by taking the natural logarithm of each ‘floating’ EMRR.
3. These intercept adjustments were then applied to the existing EMR equations for SEER local stage and for SEER regional stage. The implicit assumption here was that the proportion of local to regional breast cancer was roughly similar across all combinations of ER status/PR status/HER2 status.

All possible combinations of ER status/PR status/HER2 status were necessary for our calculations, but only the emboldened subtypes (S2 Table) were included in the model, as HER2- subtypes do not warrant treatment with trastuzumab.

**HR calibration**

In this analysis, the HR for breast cancer death should be considered interchangeable with the excess mortality risk ratio (EMRR). We allowed competing mortality from other causes varied by age and this created variation in the EMRR, which was then used for corresponding age groups. For example, the HR for overall survival, 0.63 [6], resulted in a HR for breast cancer death of 0.62 for the “40-49” age group and 0.50 for the “≥60” age group (Table 1 in manuscript).

References

1. Dickman PW, Sloggett A, Hills M, Hakulinen T. Regression models for relative survival. Statistics in Medicine. 2004;23(1):51-64.

2. National Cancer Institute. SEER Stat Fact Sheets: Breast Cancer. Bethesda: National Cancer Institute, 2013.

3. Blakely T, Costilla R, Soeberg M. Cancer Excess Mortality Rates Over 2006-2026 for ABC-CBA. Wellington: Department of Public Health, University of Otago, 2012.

4. Cianfrocca M, Goldstein LJ. Prognostic and predictive factors in early-stage breast cancer. The Oncologist. 2004;9(6):606-16.

5. Parise CA, Bauer KR, Brown MM, Caggiano V. Breast cancer subtypes as defined by the estrogen receptor (ER), progesterone receptor (PR), and the human epidermal growth factor receptor 2 (HER2) among women with invasive breast cancer in California, 1999–2004. The Breast Journal. 2009;15(6):593-602.

6. Perez EA, Romond EH, Suman VJ, Jeong J-H, Sledge G, Geyer CE, et al. Trastuzumab plus adjuvant chemotherapy for human epidermal growth factor receptor 2–positive breast cancer: planned joint analysis of overall survival from NSABP B-31 and NCCTG N9831. Journal of Clinical Oncology. 2014;32(33):3744-52.
